# Transcriptome analysis provides insights into the regulation of metabolic processes during postharvest cold storage of loquat (*Eriobotrya japonica*) fruit

**DOI:** 10.1038/s41438-019-0131-9

**Published:** 2019-04-06

**Authors:** Wenli Liu, Jing Zhang, Chen Jiao, Xueren Yin, Zhangjun Fei, Qingbiao Wu, Kunsong Chen

**Affiliations:** 1 School of Mathematical Science, Zhejiang University, Yuquan Campus, 310027 Hangzhou, P.R. China; 2 Boyce Thompson Institute, Cornell University, Ithaca, NY 14853 USA; 3 Zhejiang Provincial Key Laboratory of Horticultural Plant Integrative Biology, Zhejiang University, Zijingang Campus, 310058 Hangzhou, P.R. China; 4 USDA-ARS Robert W. Holley Center for Agriculture and Health, Ithaca, NY 14853 USA

**Keywords:** RNA sequencing, Plant stress responses

## Abstract

Loquat (*Eriobotrya japonica*) fruit accumulates lignin during postharvest storage under chilling conditions (0 °C), while low-temperature conditioning (LTC; 5 °C for 6 days followed by transfer to 0 °C) or heat treatment (HT; 40 °C for 4 h followed by transfer to 0 °C) can alleviate lignification. Here we compared transcriptome profiles of loquat fruit samples under LTC or HT to those stored at 0 °C at five time points from day 1 to day 8 after treatment. High-throughput transcriptome sequences were de novo assembled into 53,319 unique transcripts with an N50 length of 1306 bp. A total of 2235 differentially expressed genes were identified in LTC, and 1020 were identified in HT compared to 0 °C. Key genes in the lignin biosynthetic pathway, including *EjPAL2*, *EjCAD1*, *EjCAD3*, *4CL*, *COMT*, and *HCT*, were responsive to LTC or HT treatment, but they showed different expression patterns during the treatments, indicating that different structural genes could regulate lignification at different treatment stages. Coexpression network analysis showed that these candidate biosynthetic genes were associated with a number of transcription factors, including those belonging to the AP2, MYB, and NAC families. Gene ontology (GO) enrichment analysis of differentially expressed genes indicated that biological processes such as stress responses, cell wall and lignin metabolism, hormone metabolism, and metal ion transport were significantly affected under LTC or HT treatment when compared to 0 °C. Our analyses provide insights into transcriptome responses to postharvest treatments in loquat fruit.

## Loquat fruit: Investigating spoilage during cold storage

Next-generation sequencing of loquat fruit stored in different ways reveals the genes and proteins that help reduce fruit spoil. Loquat fruit become rigid and woody during cold storage, thanks to the build-up of lignin polymers in the fruit flesh. Lignification can be prevented with exposure to heat or preconditioning to cool temperatures before cold storage. Zhangjin Fei at Cornell University, New York, US, Qingbiao Wu at Zhejiang University in Hangzhou, China, and co-workers used comparative RNA sequencing to explore the molecular mechanisms inherent in lignification. The team placed three loquat groups in post-harvest cold storage for 8 days: one pre-treated with heat, one conditioned at cool temperatures, and one with no pre-treatment. They identified key genetic differences between the groups and several protein families that may regulate lignification.

## Introduction

Fruit firmness is an important fruit quality trait. How to maintain optimal fruit firmness is consistently a topic of postharvest or horticultural research. For loquat fruit, lignification is induced during low-temperature (0 °C) storage, which significantly decreases its commodity value^1,2^. Treatments, such as low-temperature conditioning (LTC; 5 °C for 6 days followed by transfer to 0 °C) and heat treatment (HT; 40 °C for 4 h followed by transfer to 0 °C), have been developed to alleviate chilling-induced lignification. However, the underlying regulatory mechanisms of these treatments have rarely been investigated.

Lignin is the main component of plant secondary cell walls and plays an essential role in many different types of cells to provide structural support for growth, development, and defense^3–5^. The biosynthetic pathway of lignin has been well studied in plants and involves the synthesis of monolignols followed by construction of lignin polymers through oxidative polymerization. The main biosynthetic route, the phenylpropanoid pathway, synthesizes the monolignols p-coumaryl, coniferyl, and sinapyl alcohol from phenylalanine. Studies in the model plant Arabidopsis have shown that cinnamate 4-hydroxylase (C4H), p-coumarate 3-hydroxylase, cinnamoyl-CoA reductase (CCR), and 4-coumarate:CoA ligase (4CL)^6–8^ play roles in regulating the lignin content, while 4CL simultaneously changes the lignin composition. Homologs of these genes have been identified in many woody trees and have been shown to have similar functions in lignin biosynthesis in these species^9,10^. Several transcription factors (TFs) involved in regulating the lignification process have also been identified, including those from the NAC and MYB families. More than nine MYBs have been reported as lignin biosynthesis-related TFs in Arabidopsis, functioning as either activators or repressors of lignin pathway gene expression^11,12^. Genome and transcriptome analyses have provided valuable information about the lignin biosynthetic pathway and its regulation in plant species ^13,14^.

Most studies on lignin focus on seedlings, roots, leaves, and stems from model plants and woody trees. However, lignin is also important in some fruits. Although most fruits only contain trace amounts of lignin, some fruits accumulate high levels of lignin in different layers, such as in loquat flesh^2^, mangosteen pericarp^15^, peach endocarp^16^, and stone cells in pear pulp^17^. In these fruits, lignification influences the quality and/or storability and thus consumer acceptance. Recent studies have shown correlations between the activities of lignin biosynthetic genes and fruit lignification. For instance, in peach fruit, the CCoAOMT (caffeoyl-CoA 3-O-methyltransferase) and C4H genes are induced in the endocarp layer^16^, and in strawberry, CCR and CAD (cinnamyl alcohol dehydrogenase) are considered to be candidate genes involved in fruit firmness^18^. Fruit lignification at low temperature has been studied in peach^19^ and zucchini fruits^20^. Loquat fruit is a good model for the scientific analysis of fruit lignification at low temperature. Some TFs have been characterized as being involved in low-temperature-related lignification in loquat fruit. These include two MYB TFs, EjMYB1 and EjMYB2, which function as an activator and a repressor, respectively^21^. EjAP2-1, belonging to the AP2/ERF family, has also been shown to play a role in the regulation of loquat fruit lignification via protein–protein interactions with EjMYB1 and EjMYB2^22^. In addition, EjAP2-1 appears to function as a mediator of EjHSF3-regulated lignin biosynthesis in loquat fruit^23^. Furthermore, EjNAC1 and EjMYB8 have also been implicated in loquat fruit lignification, although their relationships with the aforementioned TFs remain unclear^21,24^. Although progress has been made in understanding fruit lignification, there is only a very limited understanding of how the network of interacting TFs governs lignin production and deposition.

Through comparative transcriptome analysis, we identified key structural genes, as well as potential regulatory genes, associated with low-temperature-induced loquat fruit lignification. We also identified genes from fruit-quality-related biological processes that were significantly affected in loquat fruit under LTC/HT compared to fruit stored at 0 °C. This information provided novel insights into the molecular mechanisms underlying low-temperature-induced lignification and other simultaneously affected biological processes in loquat fruit.

## Materials and methods

### Plant materials and treatments

Loquat fruits (*Eriobotrya japonica* Lindl., cv. Luoyangqing) were harvested from an orchard in Luqiao, Zhejiang, China. Fruits were harvested at commercial maturity and transported to the laboratory on the day of harvest and then screened for uniform size and maturity and absence of disease and mechanical damage.

Two separate pools from the same batch of fruits underwent HT and LTC, and another pool was stored at 0 °C. Physiological changes in fruits under LTC (5 °C for 6 days followed by transfer to 0 °C for 2 days) were described in our previous report^22^. For HT, the loquat fruits were treated at 40 °C for 4 h and then transferred to 0 °C. Three biological replicates were performed for each treatment. The fleshy tissues were collected at days 0, 1, 2, 4, 6, and 8 during treatment, quickly frozen in liquid nitrogen, and stored at −80 °C until needed. To compare the different treatments, the initial day (day 0) was not included in the RNA-Seq analysis.

### Measurement of fruit firmness and lignin content

Fruit firmness and lignin content are the main indices used to monitor postharvest lignification of loquat fruit^1,2^. Fruit firmness was measured using a TA.XTplus Texture Analyser (Stable Micro Systems, UK), with a 5-mm diameter probe, a penetration rate of 1 mm s^–1^, and a penetration depth of 4 mm^22^. The firmness of each fruit was averaged from two measurements, 90° apart at the fruit equator, after removal of a small piece of peel. Fruit firmness was expressed as Newtons (N), and 10 individual fruit replicates were tested.

Lignin content was determined according to the method described by Shan et al.^25^. The frozen sample was ground into powder and homogenized in 5 ml of washing buffer (100 mM K_2_HPO_4_/KH_2_PO_4_, 0.5% Triton X-100, 0.5% PVP, pH 7.8). The mixture was cultured on a shaker at room temperature at 200 rpm for 30 min and then centrifuged (6000 × *g*, 25 °C) for 20 min. The pellet was suspended and washed twice in the washing buffer and then four times in 100% methanol. The pellet was dried at 80 °C in an oven overnight. Ten milligrams of the dry powder were dissolved in 1.0 ml of 2.0 M HCl and 0.1 ml of thioglycolic acid. The mixture was boiled in a water bath (100 °C) for 8 h and then cooled on ice for 5 min before centrifugation at 10,000 × *g* for 20 min at 4 °C. The pellet was washed with distilled water and suspended in 2.0 ml of 1.0 M NaOH. After agitating slightly at room temperature for 18 h, the solution was centrifuged at 10,000 × *g* for 20 min. The supernatant (0.5 ml) was transferred to a new tube with 0.1 ml of concentrated HCl. The tubes were left at 4 °C for 4 h to precipitate the lignin thioglycolic acid, followed by centrifugation at 10,000 × *g* for 20 min at 4 °C, and the precipitate was dissolved in 1 ml of 1.0 M NaOH. Absorbance was measured at 280 nm using 1.0 M NaOH as the blank. Data were expressed on a fresh weight basis, and all measurements were done in triplicate.

### RNA-Seq library preparation

Total RNA was extracted from the fleshy tissues using the QIAGEN RNeasy Plant Mini Kit according to the manufacturer’s instructions (QIAGEN, California, USA). RNA quality was evaluated via electrophoresis on 1% agarose gels, and RNA quantity was determined by a NanoDrop 1000 spectrophotometer (Thermo Scientific, Wilmington, DE, USA). Strand-specific RNA-Seq libraries were constructed using the protocol described in Zhong et al.^26^ and sequenced on the Illumina HiSeq 2500 platform in the single-end mode.

### RNA-Seq data processing, de novo assembly, and comparative transcriptome profiling

Raw RNA-Seq reads were first processed with Trimmomatic^27^ to trim adapter and low-quality sequences. After trimming, reads <40 bp were discarded, RNA-Seq reads were aligned to the ribosomal RNA database^28^ using Bowtie^29^, and those that could be aligned were removed. The final cleaned reads were de novo assembled into contigs using Trinity^30^ with the minimum kmer coverage set to 10. The cleaned reads were then aligned to the assembled contigs using Bowtie^29^. Contigs in which the aligned reads in the sense direction were <10% of those aligned in the antisense direction were discarded since they might be false transcripts due to incomplete digestion of the second strand during the strand-specific RNA-Seq library construction step. Contigs having no match to plant sequences but matching sequences from bacteria, fungi, viruses, or archaea in the GenBank nt database were removed. The redundancies of Trinity-assembled contigs were removed using iAssembler ^31^.

To functionally annotate the final assembled contigs, their sequences were blasted against the TrEMBL, Swiss-Prot, and Arabidopsis protein (TAIR) databases, with *E*-value cutoffs of 1E−10. Based on their hits in the three databases, gene ontology (GO) terms were assigned to the assembled contigs.

The final cleaned high-quality RNA-Seq reads were aligned to the assembled contigs using Bowtie^29^. Based on the alignments, raw counts of each contig were derived and normalized to RPKM (reads per kilobase exon model per million mapped reads). Raw counts were fed to edgeR^32^ to identify differentially expressed genes (DEGs) between LTC or HT and 0 °C with a cutoff of fold change ≥2 and false discovery rate ≤0.05. Clustering of gene expression profiles was performed using STEM^33^.

### Entropy weight method

To evaluate the importance of DEGs based on their absolute expression levels (RPKM) and relative expression levels (fold change), an entropy weight method was used. For an *m*-indicator and *n*-gene problem, the entropy of the *i*th indicator is defined as

$$H_i = \frac{1}{{\ln n}}\mathop {\sum}\limits_{j = 1}^n {f_{ij}\ln f_{ij},i = 1,2,...,m}$$

where $f_{ij} = r_{ij}/{\sum} {r_{ij}} \cdot$ Thus the weight for each entropy is

$$w_i = \frac{{1 - H_i}}{{m - \mathop {\sum}\nolimits_{i = 1}^m {H_i} }} \cdot$$

Here five expression data points under LTC (or HT) and five-fold-change data points compared to the corresponding time points under 0 °C were used to calculate the entropy and generate a sorted list of DEGs in LTC or HT. This method was implemented in MATLAB.

#### Real-time PCR analysis

Gene-specific oligonucleotide primers were designed for real-time PCR and are listed in Supplementary Table 6. The LightCycler 1.5 instrument (Roche) was used for real-time PCR. It was initiated by 5 min at 95 °C followed by 45 cycles of 95 °C for 5 s, 60 °C for 5 s, and 72 °C for 10 s. Finally, it was completed with a melting-curve analysis. The PCR mixture (10 μl total volume) contained 2 μl of 5× LightCycler FastStart DNA MasterPLUS SYBR Green I Master Mix (Roche), 0.5 μl of each primer (10 μM), 1 μl of diluted cDNA, and 6 μl of PCR-grade H_2_O. Each run included no-template controls and a melting-curve analysis.

## Results

### Regulation of loquat fruit lignification

**Fig. 1:**
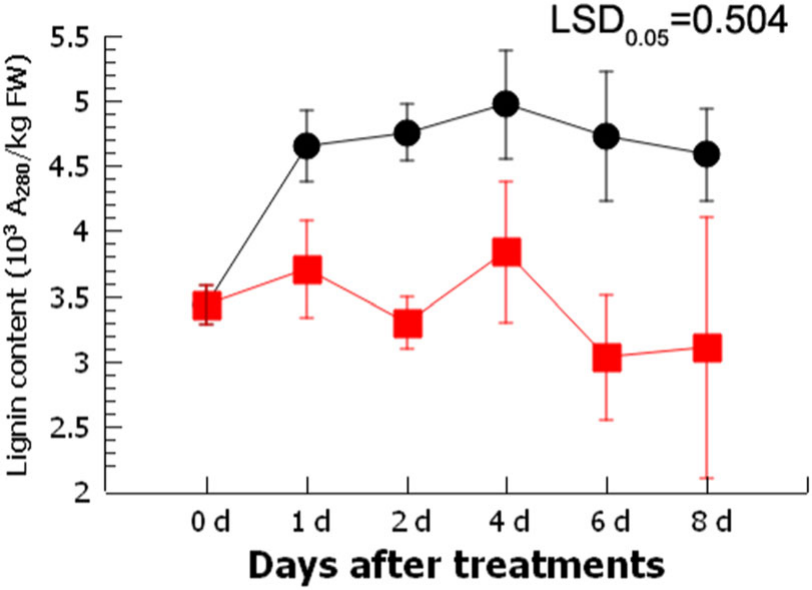
Comparison of the lignin content of loquat fruit stored at 0 °C with and without a 4-h heat treatment (HT). Error bars indicate S.E. from three replicates. LSD value represents the least significant difference at 0.05

Ripe loquat fruits (cv. Luoyangqing) were sampled and stored under three different conditions: low temperature (0 °C), HT (40 °C for 4 h followed by transfer to 0 °C), and LTC (5 °C for 6 days followed by transfer to 0 °C). The lignin content increased from 3.43 × 10^3^ A_280_/kg FW to 4.58 × 10^3^ A_280_/kg FW over a period of 8 days at 0 °C. Fruit stored under HT had a lower lignin content than fruit stored at 0 °C (Fig. 1). Similar results, showing a reduction in both lignin and firmness of loquat fruits during storage at LTC, were reported previously^22^.

### RNA-Seq, de novo assembly, and functional annotation

To obtain a better understanding of the molecular mechanisms underlying the lignin regulation of loquat fruits stored under these three different conditions, we performed comparative transcriptome analysis using RNA-Seq. A total of 48 RNA-Seq libraries were constructed and sequenced. These libraries were from samples at five or six time points for each of the three treatments (0 °C, LTC, and HT), with three biological replicates for each time point (Supplementary Table 1). After removal of adapters, low-quality sequences and ribosomal RNA reads, a total of approximately 517 million cleaned reads were obtained. Since no reference genome is available for loquat, we de novo assembled these reads into 53,319 unique transcripts with an N50 length of 1306 bp and an average length of 798 bp.

Of these 53,319 assembled transcripts, 36,252 (68.0%) were successfully annotated by homologous proteins in at least one of the three protein databases (TrEMBL, Swiss-Prot, and TAIR), and 26,382 transcripts (approximately 72.8% of the total annotated transcripts) were annotated by homologous proteins in all three databases (Supplementary Figure 1). GO terms were assigned to 35,183 (66.0%) transcripts.

### DEGs in response to HT and LTC

**Fig. 2:**
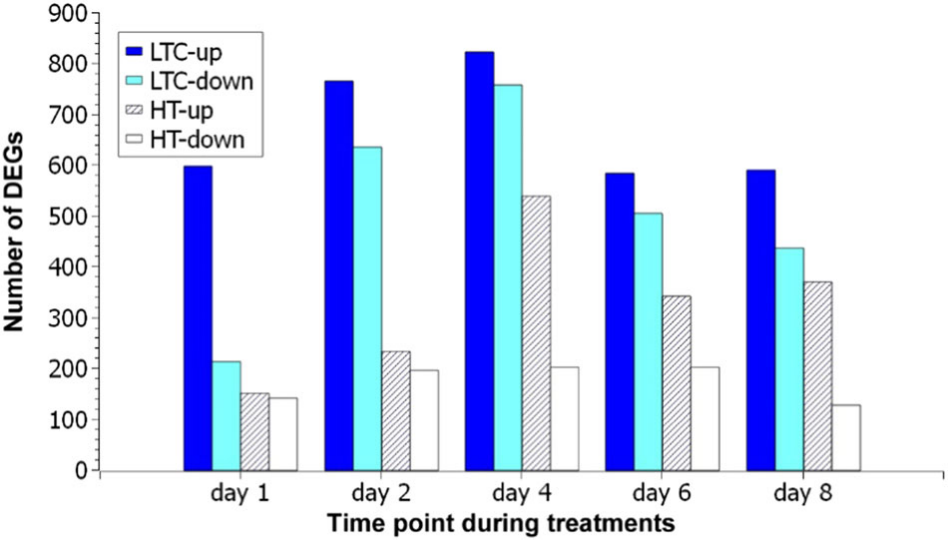
Number of differentially expressed genes at each sampling time point after low-temperature conditioning or heat treatment compared to the 0 °C condition

We identified 5824 DEGs between LTC and 0 °C samples and 3981 between HT and 0 °C samples. To generate a more focused candidate DEG set, we selected genes differentially expressed at a minimum of two of the five time points that showed the same trend (either an increase or decrease) in any of the treatments. This method reduced the DEG numbers to 2235 and 1020 in LTC vs. 0 °C and HT vs. 0 °C, respectively. The numbers of DEGs at each time point are shown in Fig. 2. The LTC treatment resulted in more DEGs than the HT treatment at all five time points, and there were more upregulated genes than downregulated ones in both LTC and HT. Both LTC and HT treatments showed the largest number of DEGs at day 4.

Genes differentially expressed throughout nearly the entire time course (from day 1 to day 8) were the most responsive to the LTC or HT treatment. Therefore, we selected genes differentially expressed at least at four time points in each treatment: 101 and 50 upregulated and 79 and 13 downregulated genes were detected in LTC and HT, respectively (Supplementary Table 2). A comparison of these genes between the two treatments was performed, and six genes appeared in both LTC and HT (Supplementary Table 3). Of these six genes, two were downregulated and annotated as encoding an alcohol dehydrogenase (*UN68301*) and an auxin-binding protein (*UN21207*). The four upregulated genes were annotated as encoding a CBL-interacting protein kinase 08 (*UN00386*), an RNA-binding protein (*UN30230*), a UDP-glucuronate decarboxylase protein 1 (*UN35156*), and a gibberellin 3-beta hydroxylase (*UN68191*).

When checking DEGs, those with both high absolute and high relative expression levels should be a priority. Here we applied an analysis on both the expression and the fold changes on all five time points (a total of ten values) under LTC or HT using the entropy weight method (see the Materials and methods section for details). The ten-dimensional data were reduced to one value, and a new sorted index was generated for the DEGs to identify the top gene list (Supplementary Table 4). Under the HT condition, genes encoding the heat shock proteins dominated the top list. After excluding these heat shock genes, the top 10 genes (Supplementary Table 5) contained some DEGs mentioned above, such as *UN00386* (CBL-CIPK) in the top list of LTC and *UN68191* (gibberellin 3-beta hydroxylase) under HT.

### Lignin and cell wall biosynthesis

#### Differentially expressed lignin-related TFs and structural genes

**Fig. 3:**
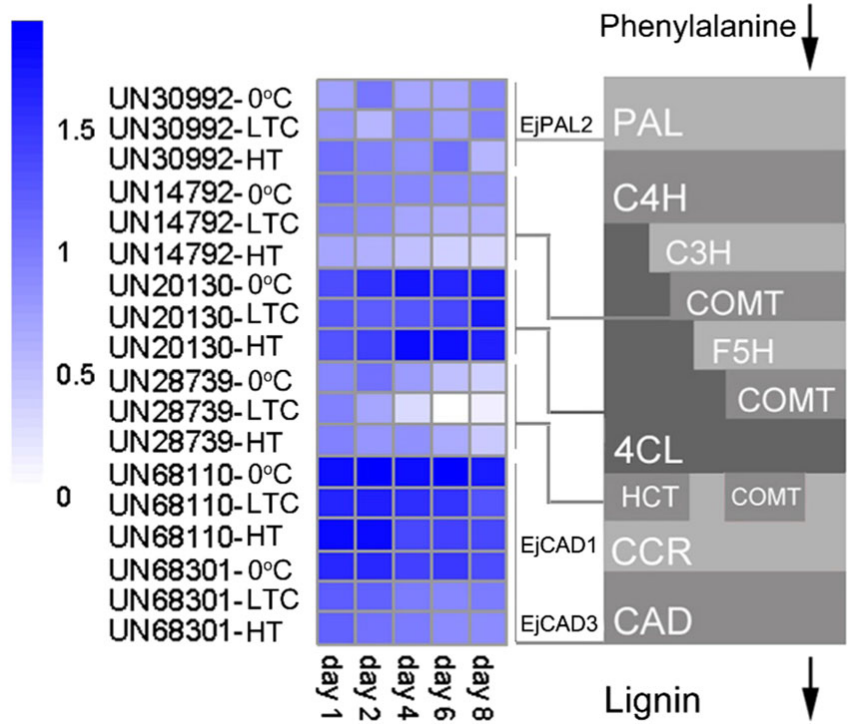
Differential expression of genes in the lignin biosynthetic pathway under low-temperature conditioning or heat treatment compared to 0 °C

**Fig. 4:**
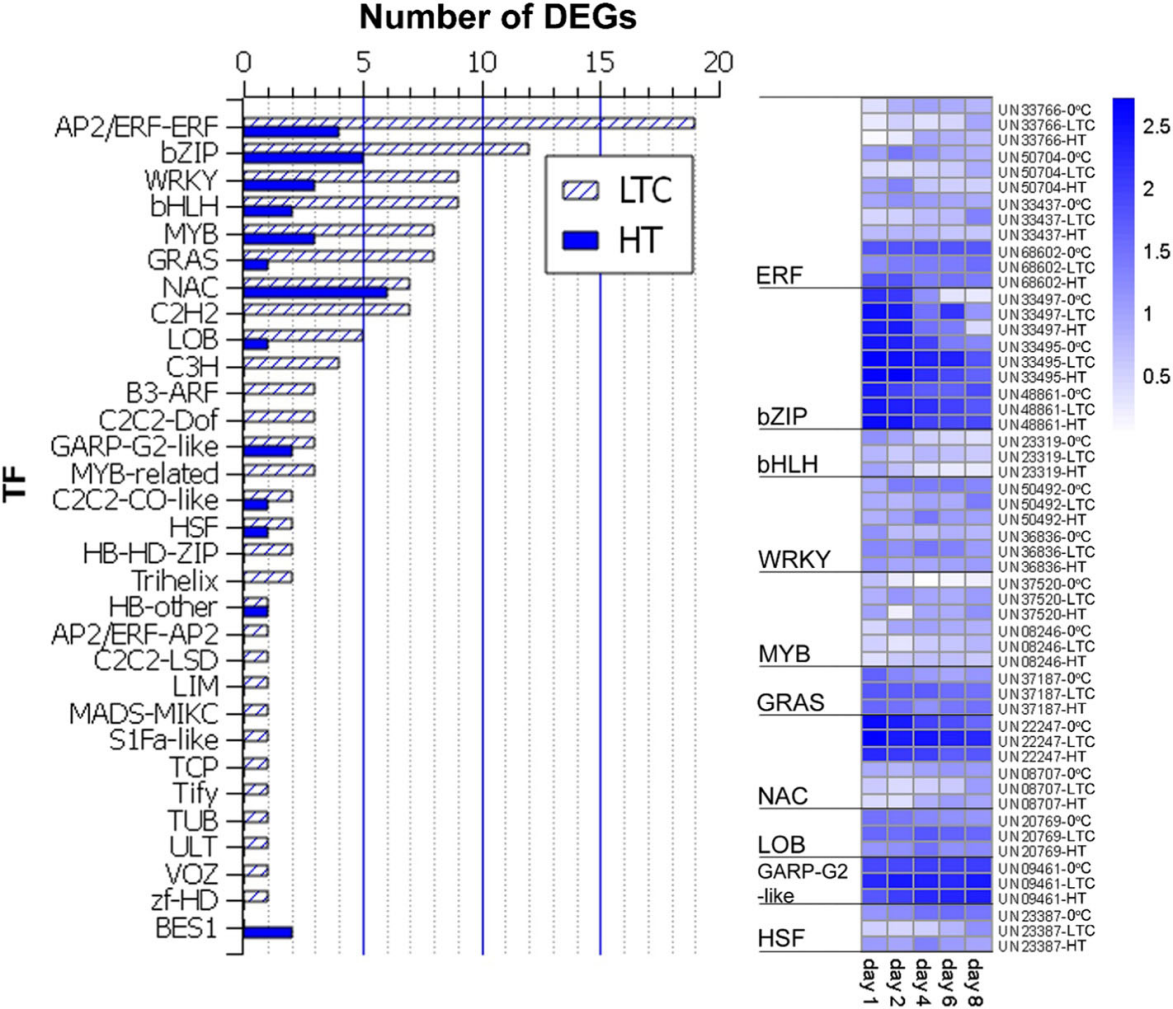
Distribution of differentially expressed transcription factors (TFs) under low-temperature conditioning (LTC) or heat treatment (HT) compared to 0 °C. Expression profiles of TFs differentially expressed under both LTC and HT are shown

Genes encoding enzymes in the lignin biosynthetic pathway have been widely studied in model plants as well as in some fruit species. In this study, six highly differentially expressed structural genes in the lignin biosynthetic pathway were identified. Except for *EjPAL2*, the other five structural genes are marked in the late steps of the pathway (Fig. 3). The quantitative reverse transcriptase–PCR validation of these candidate genes’ expression is shown in Supplementary Fig. 3. Their different expression patterns across the LTC or HT treatment indicated that multiple structural genes could act differently at different stages of chilling injury and could be candidate structural genes contributing to loquat fruit lignification at different time points. TFs play crucial roles in regulating lignin biosynthesis, as reported in model plants, woody trees, and crops^8,34^. We screened our assembled transcripts and predicted a total of 1163 TFs from 62 families, of which 120 and 32 were DEGs under LTC and HT, respectively, which were distributed in 31 families (Fig. 4). Of these TF families, the AP2/ERF family contained the most DEGs (20), followed by the bZIP family with 14 DEGs. Eighteen TFs belonging to ten families were differentially expressed under both LTC and HT.

#### Coexpression network between lignin-related TFs and structural genes

**Fig. 5:**
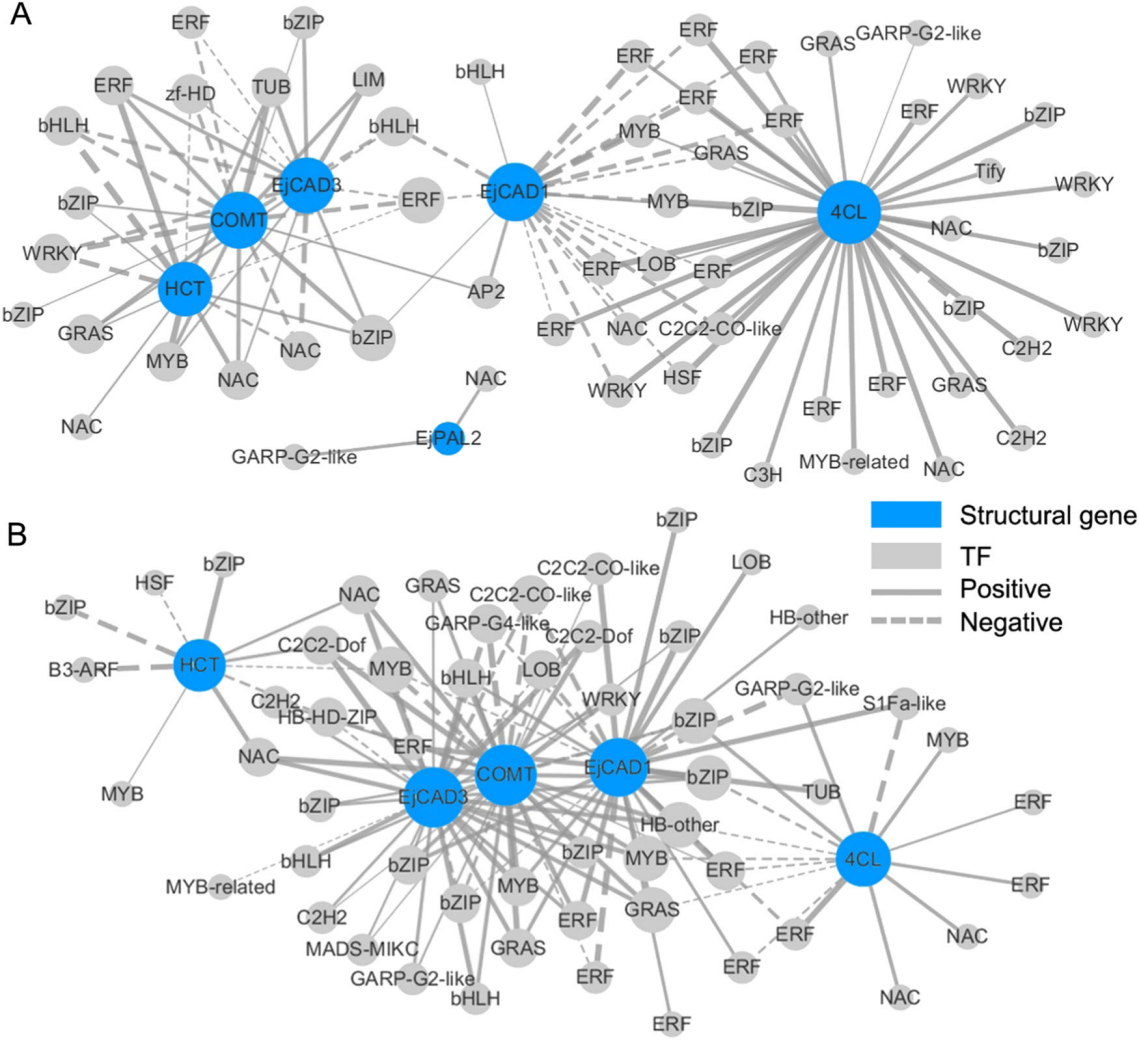
Coexpression networks between candidate structual genes and transcription factors. Coexpression networks between differentially expressed structural genes in the lignin biosynthetic pathway and differentially expressed transcription factors (TFs) under low-temperature conditioning (**a**) and heat treatment (**b**). The width of the edge between each TF node and structural gene node reflects the correlation coefficient

Using the gene expression profile data, we constructed coexpression networks between different TFs and structural genes based on the Pearson product–moment correlation coefficient. The correlation coefficient was calculated separately using the expression data under LTC and HT. Owing to the limited number of samples, we applied a very stringent cutoff for the correlation coefficient (0.9 for positive correlation and −0.9 for negative correlation). The resulting coexpression networks indicated that *EjCAD3* (*UN68301*) and *COMT* (*UN14792*) were highly correlated since most of their connected TFs were in common in the LTC and HT samples (over 80% of all their connected TF nodes) (Fig. 5). Both CAD and COMT catalyze the final steps of the lignin biosynthesis. Their connected TFs included an ERF (*UN48864*), an NAC (*UN68419*), a bZIP (*UN48861*), and an MYB (*UN68719*). Our DEG analysis showed that *UN68419* and *UN68719* were differentially expressed at the early time points under LTC or HT, and *UN48861* showed high expression levels throughout the entire time course. The HCT (hydroxycinnamoyl transferase) (*UN28739*) also shared many connected TFs with *EjCAD3* and COMT under LTC, while under HT, *EjCAD1* (*UN68110*) shared more TFs connected to *EjCAD3* and COMT. The highly differentially expressed AP2 (*UN33535*) and MYB (*UN19253*) genes were also highly correlated with *EjCAD3* and COMT under the HT condition.

#### Cell wall metabolism-related DEGs

**Fig. 6:**
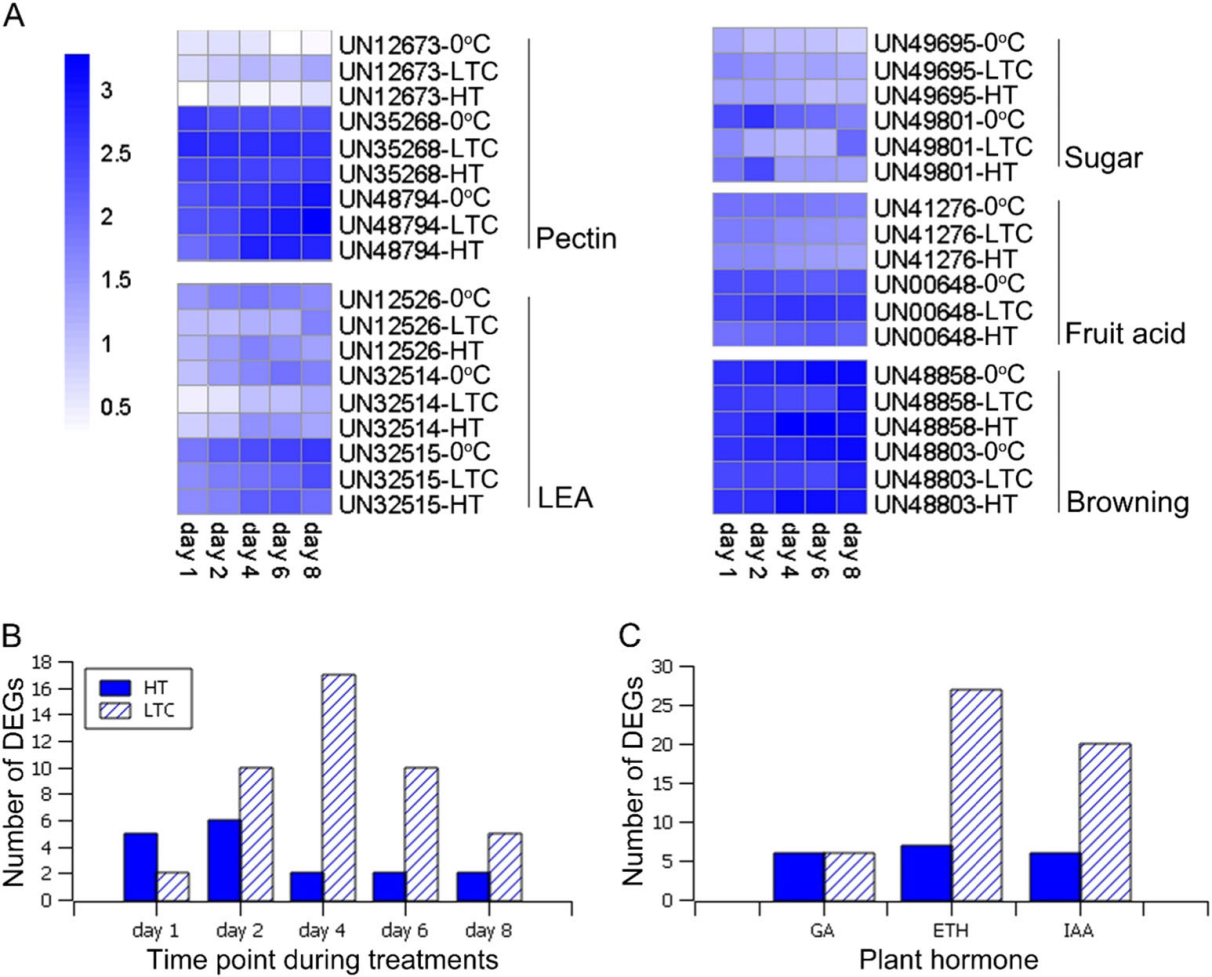
Differential expression of LEA (late embryogenesis abundant) and hormone-related genes. **a** Gene expression heat map of LEA genes and filtered fruit-quality-related genes under low-temperature conditioning (LTC), heat treatment (HT), and 0 °C. **b** Numbers of differentially expressed LEA genes under LTC or HT at different time points compared to 0 °C. **c** Numbers of differentially expressed plant-hormone-related genes under LTC or HT compared to 0 °C

Genes involved in the biosynthesis of different components of the cell wall other than lignin, including pectin, cellulose, and xylans, were also found to be responsive to LTC or HT treatment. Pectin was the most obviously affected in terms of gene expression changes. We obtained three DEGs related to pectin metabolism (Supplementary Tables 2 and 4): *UN12673* (encoding a pectate lyase) and *UN35268* (encoding a pectinacetylesterase) that were upregulated in LTC and *UN48794* (encoding a pectinesterase) that was first downregulated and then upregulated throughout the time course in HT (Fig. 6a).

#### GO term enrichment analysis

We further performed GO term enrichment analysis and identified lignin-/cell-wall-related biological processes enriched in DEGs under both LTC and HT. These processes included cell-wall-related processes, lignin metabolic processes, and phenylpropanoid biosynthetic processes (Table 1). The detailed relationship between the enriched lignification-related biological processes under LTC and HT is shown in Supplementary Fig. 2.

**Table 1:** Enriched GO terms related to lignification, cell wall metabolism, and abiotic stress responses under LTC and HT

GO ID	GO annotation	Corrected *P* (LTC)	Corrected *P* (HT)
GO:0009414	Response to water deprivation	5.695E−13	1.115E−06
GO:0009409	Response to cold	2.365E−09	0.0007
GO:0009408	Response to heat	7.346E−06	0.007
GO:0071555	Cell wall organization	3.262E−06	3.732E−08
GO:0042545	Cell wall modification	0.0105	0.0388
GO:0052386	Cell wall thickening	0.0133	0.0046
GO:2000652	Regulation of secondary cell wall biogenesis	0.0064	0.0281
GO:0009808	Lignin metabolic process	0.0001	0.0038
GO:1901141	Regulation of lignin biosynthetic process	0.0001	0.011
GO:0009699	Phenylpropanoid biosynthetic process	0.0006	0.0026
GO:0009809	Lignin biosynthetic process	0.0047	0.0079
GO:0046274	Lignin catabolic process	0.0047	0.0037
GO:0070588	Calcium ion transmembrane transport	0.0001	0.0284
GO:0035584	Calcium-mediated signaling using intracellular calcium source	0.024	0.0299
GO:0030001	Metal ion transport	0.0017	0.0041
GO:0015691	Cadmium ion transport	0.0074	0.0299
GO:0042445	Hormone metabolic process	0.0139	0.0286
GO:0042304	Regulation of fatty acid biosynthetic process	0.0159	0.0328

*GO* gene ontology, *HT* heat treatment, *LTC* low-temperature conditioning

#### Gene expression patterns

**Fig. 7:**
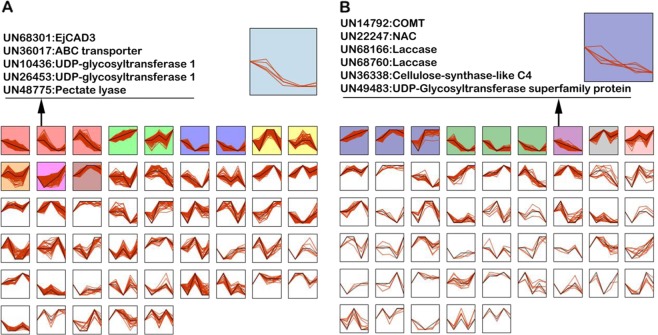
Gene expression data clustering. Expression profile clustering of genes differentially expressed under low-temperature conditioning (LTC; **a**) or high temperature (HT; **b**) compared to 0 °C. Clusters with colored backgrounds have a statistically significant number of differentially expressed genes assigned vs. the number expected. The black line in each box is the extracted expression pattern for each cluster. Genes in the same group with *EjCAD3* under LTC and COMT under HT, which were also identified by the gene ontology annotation analysis, are listed

Gene expression clustering was applied to identify genes with similar expression patterns. All the DEGs were clustered into 50 different groups (Fig. 7). Genes in the same clusters are very likely involved in the same biological processes. Therefore, once we confirm the function of a gene in the cluster, other genes in the same cluster could be considered to have similar functions. For the two identified structural genes in lignin biosynthesis, *EjCAD3* (*UN68301*) and *COMT* (*UN14792*), we found several genes in the same cluster that were also identified to be in the same group by GO annotation analysis (Fig. 7), further supporting their potential roles in lignin biosynthesis.

### Other affected biological processes

In-depth analysis of the filtered DEG sets (Supplementary Tables 2 and 4) identified genes involved in interesting biological processes other than lignin-/cell-wall-related ones under the LTC or HT treatment compared to 0 °C. These biological processes included those related to stress responses (including responses to cold, heat, and water deprivation), regulation of fatty acid biosynthetic processes, hormone metabolic processes, and several metal-ion-related processes (mainly calcium and cadmium transport), all of which were significantly enriched in the DEG sets.

#### Stress responses

CBL-CIPKs and LEA (late embryogenesis abundant) genes have been reported to be induced by cold stress^35^. A number of differentially expressed LEA genes were identified, and the number of these LEA genes at each time point is shown in Fig. 6b. Under HT, more LEA genes were differentially expressed at the early time points (day 1 and day 2). In addition, we identified three LEA genes that were differentially expressed at day 1/day 2 under both LTC and HT treatments, and their expression levels were decreased under both LTC and HT compared to 0 °C (Fig. 6a). Furthermore, a number of heat-shock-related genes were also identified under HT; as expected, they were all upregulated.

#### Hormone metabolic processes

Plant hormones are highly correlated with stress responses under temperature-based postharvest treatments. Changes in plant hormone contents can affect fruit quality. A number of plant-hormone-related genes were responsive to HT or LTC in loquat fruit. The numbers of DEGs related to gibberellin (GA), ethylene (ETH), and auxin (IAA) are shown in Fig. 6c. LTC affected the expression of more IAA- and ETH-related genes than HT, while the expression of a similar number of GA-related genes was affected by LTC and HT.

#### Enzymatic browning

Enzymatic browning is one of the symptoms of loquat fruit chilling injury, which is usually displayed after the fruit are transferred from cold storage to room temperature. Enzymatic browning was not observed in the present study due to the short-term storage. However, the transcriptome analysis indicated that two enzymatic-browning-related polyphenol oxidase genes, *UN48803* and *UN48858*, were downregulated under LTC throughout the entire time course (Fig. 6a), which may contribute to the lower browning index in LTC-treated fruits^1^.

#### Sugar- and acid-related genes

In loquat fruit, fructose, sucrose, glucose, and sorbitol are the four main sugar compounds correlated with fruit taste. From the DEG analysis, we identified the gene *UN49695* (encoding a fructokinase) that was upregulated under LTC but not differentially expressed under HT, and another gene, *UN49801* (encoding a sorbitol dehydrogenase), that was downregulated under both LTC and HT (Fig. 6a). For fruit acidity, *UN00648* (encoding a malic acid enzyme) and *UN41276* (encoding a fruit-acidity-related protein) were found to be downregulated under HT, while under LTC, *UN00648* was upregulated (Fig. 6a). These results indicated that LTC and HT could affect fruit sugar- and acid-related genes, which would eventually influence the fruit flavor, although LTC and HT could have different impacts.

## Discussion

Loquat fruit is one of the best systems for fleshy fruit lignification research^21,23,36^. Here we performed deep transcriptome sequencing of 48 loquat fruit samples under three different postharvest storage conditions using RNA-Seq. The transcriptome sequences were de novo assembled into 53,319 transcripts, of which 70.2% were annotated. In addition to a recently reported loquat transcriptome assembly by Song et al.^37^, our assembled and annotated loquat transcripts provide a valuable resource for loquat research and improvement.

The primary purpose of the present study was to compare gene expression patterns in loquat fruits stored at 0 °C after an HT or LTC treatment, both of which cause significantly reduced lignification, in order to identify structural and regulatory genes that contribute to loquat fruit lignification during cold storage. Some studies have focused on structural genes to understand the lignin regulation pathway in fruits, such as CCR in pear^38^ and 4CL in mulberry^39^. Here we have also identified several structural genes, such as those encoding PAL, 4CL, COMT, HCT, and CAD, that contribute to the lignification of loquat fruits during cold storage (Fig. 4). We identified the PAL gene *EjPAL2*, which we have previously shown is involved in phenylpropanoid synthesis during early stages of fruit development when there is a considerable increase in vascular tissues^25^. Phenylpropanoid synthesis is a very early step of lignin biosynthesis, and low temperature mainly causes damage to the vascular tissues in the early stages of fruit development. Therefore, the upregulated expression of *EjPAL2* could reinforce the vascular tissues for improved low-temperature tolerance. The *HCT* and *4CL* genes were downregulated under both LTC and HT at day 1/day 2, while *COMT* and *EjCAD3* were downregulated at all sampled time points under both treatments. It is also notable that the *HCT* and *4CL* genes have more significant expression differences under LTC compared to 0 °C than under HT, while *EjPAL2* and *COMT* have more significant expression differences under HT compared to 0 °C than under LTC. Thus these results indicated that multiple structural genes are involved in fruit lignification; however, similar repression effects of LTC and HT treatment may have distinct main targets. As mentioned above, *COMT* and *EjCAD3* were highly correlated. The *COMT* gene merits a more in-depth functional analysis since there is no report on *COMT* genes regulating loquat fruit lignification, while evidence has shown the importance of *COMT* in lignin biosynthesis in Arabidopsis^40,41^. Collectively, our results indicate that various lignin biosynthetic genes contribute to loquat fruit lignification during cold storage.

Studies in model plant species have shown that lignin biosynthesis is regulated by multiple TFs, such as those in the NAC and MYB families^12,42^. Our previous studies also showed that several TFs, including *EjMYB1/2*^21^, *EjMYB8*^24^, and *EjAP2-1*^22^, are involved in the regulation of loquat fruit lignification. In the present study, RNA-Seq analysis identified a total of 134 TFs in loquat fruit belonging to 31 families that were responsive to either LTC or HT. The previously reported TFs, which include *EjAP2-1* (*UN17693*)^22^ and three MYB TF genes including *EjMYB8* (*UN08246*)^24^, are among the 134 identified TFs. In addition to these previously reported TFs, other TFs that showed similar responses to LTC and HT, such as the NAC gene (*UN68419*), are also candidate regulators of loquat fruit lignification, although their exact functions require future analyses. The NAC gene (*UN22247*) identified from expression clustering, which appears in the coexpression networks of HT, and another NAC gene *(UN04037*) correlated with *EjPAL2* under LTC are also candidate regulators. Our previous study indicated that *EjNAC3*^43^ directly binds to the *EjCAD1*-like promoter. Based on the RNA-Seq results, this NAC (*UN04037*) may regulate fruit lignification via *EjPAL2*, which may reveal a novel mechanism for NAC regulation of lignification. The present omics-based analyses have expanded the view of possible TF activities in loquat fruit. The predicted correlations between TFs and structural genes have given us useful information that can guide us in the performance of more in-depth analyses of loquat fruit lignification induced by chilling injury.

Unlike the lignin biosynthetic genes, other functional genes involved in fruit lignification have rarely been studied. For instance, there are three LEA genes downregulated by both LTC and HT (Fig. 6a), which are also positively correlated with loquat fruit lignin content. These LEA genes showed similar responses to both LTC and HT treatments, especially in the early stage (day 1 and day 2), which may be the main stress-related genes in loquat lignification. However, in loquat fruit, relationships between LEA genes and lignification have not been reported. The roles of LEA in lignin biosynthesis have been rarely reported in other plants, but when *IbLEA14* was overexpressed, sweet potato calli had higher lignin contents of monolignol and the expression of related genes^44^. Other notable information came from our GO enrichment analysis. Although many DEGs were annotated as being related to the cold response and lignin biosynthesis, as expected, a group of metal-ion-related genes were also differentially expressed. The correlation of metal ion and cold responses have been widely studied in other plant species, and it has been reported that the increased cold tolerance of wheat leaves occurs not only at a low hardening temperature (4 °С) but also in the presence of cadmium^45^. In addition, cadmium treatment can increase the activity of PAL, an important enzyme in lignin biosynthesis^46^. There are also research findings on the effect of Ca^2+^ on the cell wall in fruit^19,47^. Divalent metal ions contribute to the cold resistance of the cell wall in fruit, and the dynamic equilibrium of the ion-binding state in the cell wall is affected by the temperature and the ion concentration in the environment. These genes, as well as some others, have rarely been studied in relation to loquat fruit lignification but may contribute to it.

## Conclusions

Loquat fruits accumulate high amounts of lignin during postharvest storage under chilling conditions, which substantially decreases their quality and storability and thus consumer acceptance. Loquat fruits treated with LTC or HT showed reduced lignification. By comparing the transcriptome profiles of loquat fruits at different time points under LTC or HT to those under chilling (0 °C), we identified a large number of DEGs and found that biological processes such as stress responses, cell wall and lignin metabolism, hormone metabolic processes, and metal ion transport were significantly affected by LTC or HT compared to 0 °C. Key genes in the lignin biosynthetic pathway that were differentially expressed under LTC or HT compared to 0 °C were identified, which may contribute directly to the regulation of lignification in loquat fruit under chilling. A number of TFs showed differential expression under LTC or HT compared to 0 °C, and their expression profiles were highly correlated with those of the key lignin biosynthetic genes. These TFs included *EjMYB1/2*^18^, *EjMYB8*^23^, and *EjAP2-1*^21^, which were reported previously to be involved in the regulation of loquat fruit lignification. Further functional studies are needed to elucidate the regulatory roles of the newly identified TFs in loquat fruit lignification. The findings of this work contribute to a better understanding of the molecular mechanisms underlying the lignification process during the postharvest storage of loquat fruit under chilling conditions.

## Supplementary information

loquat_RNAseq_Supplemental_figures

loquat_RNAseq_Supplemental_tables

## Data Availability

The raw RNA-Seq data have been deposited into the NCBI Sequence Read Archive (SRA) under the accession number SRP128075. This Transcriptome Shotgun Assembly project has been deposited at DDBJ/EMBL/GenBank under the accession number GGEW00000000. The version described in this paper is the first version, GGEW01000000. The assembled transcriptome sequences can also be downloaded from http://www.ppili.xyz/loquat/download.html.

## References

[1] Cai C (2006). Effect of 1-MCP on postharvest quality of loquat fruit. Postharvest Biol. Technol..

[2] Cai C, Xu C, Li X, Ferguson I, Chen K (2006). Accumulation of lignin in relation to change in activities of lignification enzymes in loquat fruit flesh after harvest. Postharvest Biol. Technol..

[3] Tucker G (2017). Ethylene† and fruit softening. Food Qual. Saf..

[4] Rogers LA, Campbell MM (2004). The genetic control of lignin deposition during plant growth and development. New Phytol..

[5] Vance CP, Kirk TK, Sherwood RT (1980). Lignification as a mechanism of disease resistance. Annu. Rev. Phytopathol..

[6] Vanholme R, Demedts B, Morreel K, Ralph J, Boerjan W (2010). Lignin biosynthesis and structure. Plant Physiol..

[7] Zhao Q, Dixon RA (2011). Transcriptional networks for lignin biosynthesis: more complex than we thought?. Trends Plant. Sci..

[8] Zhong R, Ye ZH (2009). Transcriptional regulation of lignin biosynthesis. Plant Signal. Behav..

[9] Neutelings G (2011). Lignin variability in plant cell walls: contribution of new models. Plant Sci..

[10] Chiang VL (2006). Monolignol biosynthesis and genetic engineering of lignin in trees, a review. Environ. Chem. Lett..

[11] Newman LJ, Perazza DE, Juda L, Campbell MM (2004). Involvement of the R2R3-MYB, AtMYB61, in the ectopic lignification and dark-photomorphogenic components of the det3 mutant phenotype. Plant J..

[12] Zhou J, Lee C, Zhong R, Ye ZH (2009). MYB58 and MYB63 are transcriptional activators of the lignin biosynthetic pathway during secondary cell wall formation in Arabidopsis. Plant Cell.

[13] Shen X (2014). Transcriptomic profiling revealed an important role of cell wall remodeling and ethylene signaling pathway during salt acclimation in Arabidopsis. Plant Mol. Biol..

[14] Carocha V (2015). Genome-wide analysis of the lignin toolbox of Eucalyptus grandis. New Phytol..

[15] Kamdee C (2014). Regulation of lignin biosynthesis in fruit pericarp hardening of mangosteen (Garcinia mangostana L.) after impact. Postharvest Biol. Technol..

[16] Dardick CD (2010). Stone formation in peach fruit exhibits spatial coordination of the lignin and flavonoid pathways and similarity to Arabidopsis dehiscence. BMC Biol..

[17] Cai Y (2010). Study of the structure and biosynthetic pathway of lignin in stone cells of pear. Sci. Hortic. (Amst.).

[18] Salentijn EMJ, Aharoni A, Schaart JG, Boone MJ, Krens FA (2003). Differential gene expression analysis of strawberry cultivars that differ in fruit-firmness. Physiol. Plant..

[19] Lu G, Li Z, Zhang X, Wang R, Yang S (2015). Expression analysis of lignin-associated genes in hard end pear (*Pyrus pyrifolia* Whangkeumbae) and its response to calcium chloride treatment conditions. J. Plant Growth Regul..

[20] Carvajal F, Palma F, Jamilena M, Garrido D (2015). Cell wall metabolism and chilling injury during postharvest cold storage in zucchini fruit. Postharvest Biol. Technol..

[21] Xu Q (2014). Activator- and repressor-type MYB transcription factors are involved in chilling injury induced flesh lignification in loquat via their interactions with the phenylpropanoid pathway. J. Exp. Bot..

[22] Zeng J (2015). EjAP2-1, an AP2/ERF gene, is a novel regulator of fruit lignification induced by chilling injury, via interaction with EjMYB transcription factors. Plant Biotechnol. J..

[23] Zeng J (2016). Regulation of loquat fruit low temperature response and lignification involves interaction of heat shock factors and genes associated with lignin biosynthesis. Plant Cell Environ..

[24] Wang, W. et al. EjMYB8 transcriptionally regulates flesh lignification in loquat fruit. *PLoS ONE* **11**, e0154399 (2016). 10.1371/journal.pone.0154399 PMC4844104 27111303

[25] Shan LL (2008). Characterization of cDNAs associated with lignification and their expression profiles in loquat fruit with different lignin accumulation. Planta.

[26] Zhong S (2011). High-throughput illumina strand-specific RNA sequencing library preparation. Cold Spring Harb. Protoc..

[27] Bolger AM, Lohse M, Usadel B (2014). Trimmomatic: a flexible trimmer for Illumina sequence data. Bioinformatics.

[28] Quast C (2013). The SILVA ribosomal RNA gene database project: improved data processing and web-based tools. Nucleic Acids Res..

[29] Langmead B, Trapnell C, Pop M, Salzberg SL (2009). Ultrafast and memory-efficient alignment of short DNA sequences to the human genome. Genome Biol..

[30] Grabherr, M. G. et al. Full-length transcriptome assembly from RNA-Seq data without a reference genome. *Nat. Biotechnol*. **29**, 644–652 (2011). 10.1038/nbt.1883 PMC3571712 21572440

[31] Zheng Y, Zhao L, Gao J, Fei Z (2010). iAssembler: a package for de novo assembly of Roche-454/Sanger transcriptome sequences. BMC Bioinformatics.

[32] Robinson MD, McCarthy DJ, Smyth GK (2010). edgeR: a Bioconductor package for differential expression analysis of digital gene expression data. Bioinformatics.

[33] Ziv BJ, Jason E (2006). STEM: a tool for the analysis of short time series gene expression data. BMC Bioinformatics.

[34] Zhao Q (2010). An NAC transcription factor orchestrates multiple features of cell wall development in *Medicago truncatula*. Plant J..

[35] Chinnusamy, V., Zhu, J.-K. & Sunkar, R. Gene Regulation During Cold Stress Acclimation in Plants. In: Sunkar R. (eds) Plant Stress Tolerance. *Methods in Molecular Biology (Methods and Protocols)*. **639**, 39–55 (2010). 10.1007/978-1-60761-702-0_3 PMC3064467 20387039

[36] Cai C (2006). Low temperature conditioning reduces postharvest chilling injury in loquat fruit. Postharvest Biol. Technol..

[37] Song, H. et al. Comparative transcriptional analysis of loquat fruit identifies major signal networks involved in fruit development and ripening process. *Int. J. Mol. Sci*. **17**, 1837 (2016). 10.3390/ijms17111837 PMC5133838 27827928

[38] Lu XP, Liu YZ, An JC, Hu HJ, Peng SA (2011). Isolation of a cinnamoyl CoA reductase gene involved in formation of stone cells in pear (*Pyrus pyrifolia*). Acta Physiol. Plant.

[39] Wang CH (2016). Characterization and functional analysis of 4-coumarate:CoA ligase genes in mulberry. PLoS ONE.

[40] Vanlholme R (2010). Engineering traditional monolignols out of lignin by concomitant up-regulation of F5H1 and down-regulation of COMT in Arabidopsis. Plant J..

[41] Moinuddin SGA (2010). Insights into lignin primary structure and deconstruction from *Arabidopsis thaliana* COMT (caffeic acid O-methyl transferase) mutant Atomt1. Org. Biomol. Chem..

[42] Nakano, Y., Yamaguchi, M., Endo, H., Rejab, N. A. & Ohtani, M. NAC-MYB-based transcriptional regulation of secondary cell wall biosynthesis in land plants. *Front. Plant Sci*. **6**, 288 (2015). 10.3389/fpls.2015.00288 PMC4419676 25999964

[43] Ge H, Zhang J, Zhang Y (2017). EjNAC3 transcriptionally regulates chilling-induced lignification of loquat fruit via physical interaction with an atypical CAD-like gene. J. Exp. Bot..

[44] Park SC (2011). Sweetpotato late embryogenesis abundant 14 (IbLEA14) gene influences lignification and increases osmotic- and salt stress-tolerance of transgenic calli. Planta.

[45] Venzhik, V., Talanova, V. V., Titov, A. F. & Kholoptseva, E. S. Similarities and differences in wheat plant responses to low temperature and cadmium. *Biol Bull Russ Acad Sci*. **42**: 508–514 (2015). 26852479

[46] Pawlak-Sprada S, Arasimowicz-Jelonek M, Podgórska M, Deckert J (2011). Activation of phenylpropanoid pathway in legume plants exposed to heavy metals. Part I. Effects of cadmium and lead on phenylalanine ammonia-lyase gene expression, enzyme activity and lignin content. Acta Biochim. Pol..

[47] Figueroa CR, Opazo MC, Vera P, Arriagada O, Díaz M, Moya-León MA (2012). Effect of postharvest treatment of calcium and auxin on cell wall composition and expression of cell wall-modifying genes in the Chilean strawberry (*Fragaria chiloensis*) fruit. Food Chem..

