# Supplementary material for: Transcriptome analysis provides insights into the regulation of metabolic processes during postharvest cold storage of loquat (Eriobotrya japonica) fruit
Source: Hortic Res. 2019 Apr 6;6:49. doi: 10.1038/s41438-019-0131-9 (PMC6441654; doi:10.1038/s41438-019-0131-9)
Supplement: Supplementary file 1 — loquat_RNAseq_Supplemental_figures [file 41438_2019_131_MOESM1_ESM.docx]

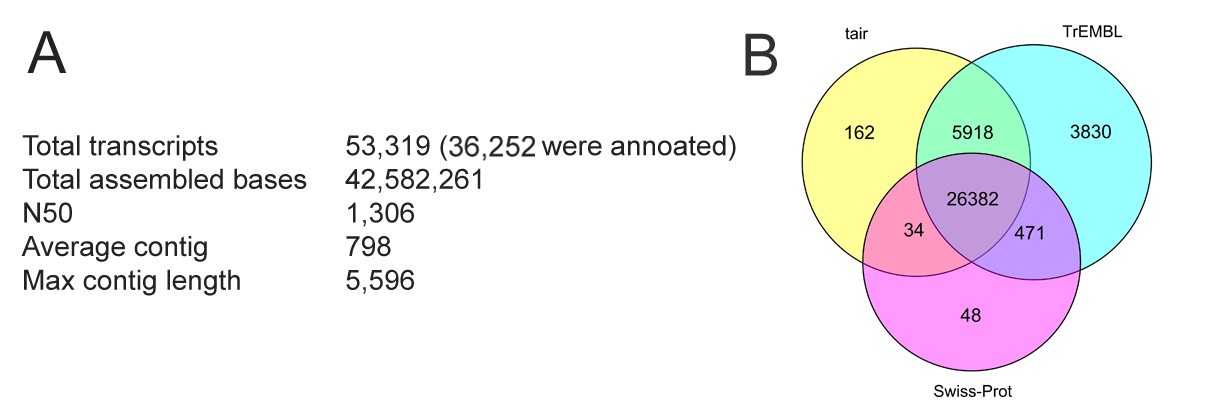


**Supplemental Figure 1:** De novo transcriptome assembly of loquat fruit. (A) Summary of the assembly. (B) Venn diagram showing numbers of assembled transcripts annotated by three databases (TrEMBL, Swiss-Prot and TAIR).


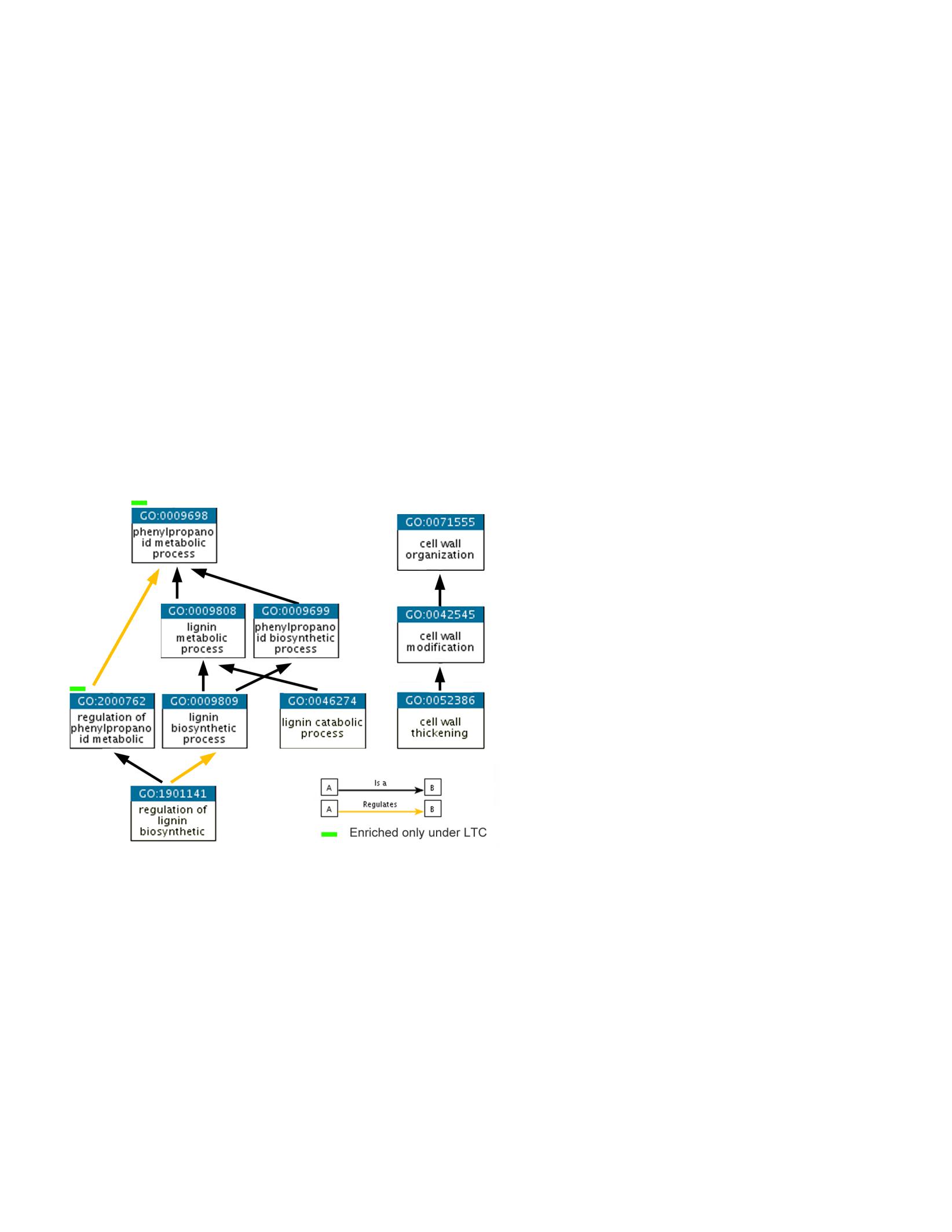


**Supplemental Figure 2:** Relationship between the enriched lignification-related gene ontology (GO) terms under LTC and HT.


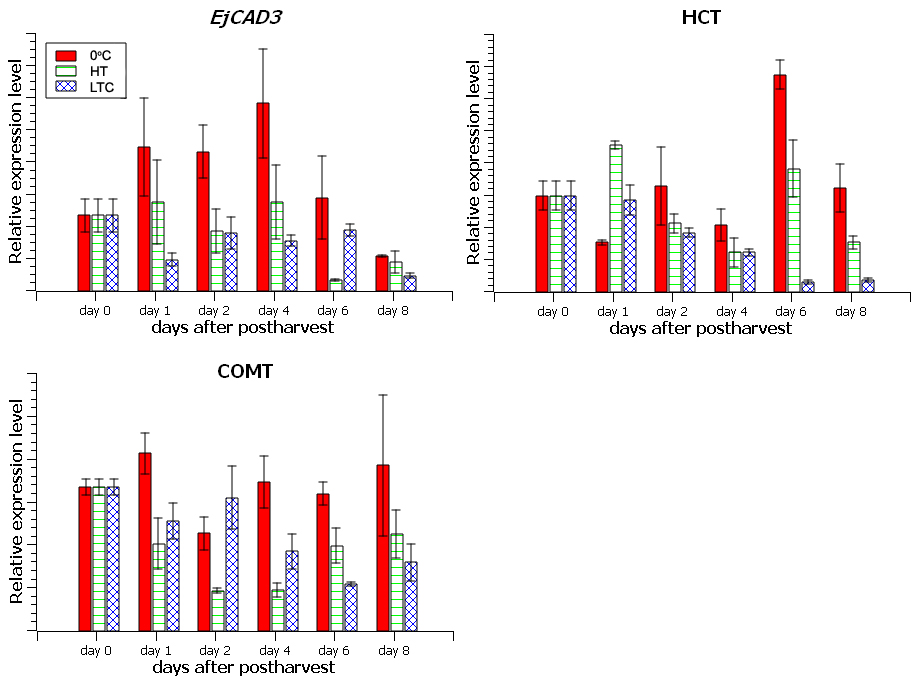


**Supplemental Figure 3:** The qRT-PCR validation of the candidate structural gene expression.
