# Supplementary material for: Transcriptome analysis provides insights into the regulation of metabolic processes during postharvest cold storage of loquat (Eriobotrya japonica) fruit
Source: Hortic Res. 2019 Apr 6;6:49. doi: 10.1038/s41438-019-0131-9 (PMC6441654; doi:10.1038/s41438-019-0131-9)
Supplement: Supplementary file 2 — loquat_RNAseq_Supplemental_tables [file 41438_2019_131_MOESM2_ESM.docx]

**Supplemental Table 1**: Summary statistics of RNA-Seq data.

| ID | treatment | Days after treatment | Clean reads | Mapped reads |
| --- | --- | --- | --- | --- |
| L-1-A | LYQ-LTC-1d-A | low temperature condition, 1 d | 9,738,305 | 7,327,285 |
| L-1-B | LYQ-LTC-1d-B |  | 8,078,076 | 6,125,500 |
| L-1-C | LYQ-LTC-1d-C |  | 13,673,346 | 10,359,426 |
| L-2-A | LYQ-LTC-2d-A | low temperature condition, 2 d | 12,867,291 | 9,806,618 |
| L-2-B | LYQ-LTC-2d-B |  | 7,844,743 | 5,966,435 |
| L-2-C | LYQ-LTC-2d-C |  | 7,719,522 | 5,898,674 |
| L-4-A | LYQ-LTC-4d-A | low temperature condition, 4 d | 7,759,397 | 5,297,501 |
| L-4-B | LYQ-LTC-4d-B |  | 10,876,478 | 8,431,165 |
| L-4-C | LYQ-LTC-4d-C |  | 6,935,180 | 5,360,378 |
| L-6-A | LYQ-LTC-6d-A | low temperature condition, 6 d | 8,906,578 | 6,857,256 |
| L-6-B | LYQ-LTC-6d-B |  | 10,261,256 | 6,360,951 |
| L-6-C | LYQ-LTC-6d-C |  | 13,575,610 | 10,374,549 |
| L-8-A | LYQ-LTC-8d-A | low temperature condition, 8 d | 10,764,335 | 8,183,107 |
| L-8-B | LYQ-LTC-8d-B |  | 7,337,449 | 5,570,300 |
| L-8-C | LYQ-LTC-8d-C |  | 9,056,220 | 7,056,602 |
| H-1-A | LYQ-HT-1d-A | heat treatment, 1 d | 9,682,678 | 7,304,058 |
| H-1-B | LYQ-HT-1d-B |  | 6,559,342 | 4,845,226 |
| H-1-C | LYQ-HT-1d-C |  | 6,008,032 | 4,499,823 |
| H-2-A | LYQ-HT-2d-A | heat treatment, 2 d | 5,933,262 | 4,458,736 |
| H-2-B | LYQ-HT-2d-B |  | 9,402,379 | 7,179,092 |
| H-2-C | LYQ-HT-2d-C |  | 11,601,704 | 8,749,684 |
| H-4-A | LYQ-HT-4d-A | heat treatment, 4 d | 11,855,919 | 9,019,166 |
| H-4-B | LYQ-HT-4d-B |  | 12,175,564 | 9,183,893 |
| H-4-C | LYQ-HT-4d-C |  | 12,937,687 | 9,763,731 |
| H-6-A | LYQ-HT-6d-A | heat treatment, 6 d | 11,625,846 | 8,857,352 |
| H-6-B | LYQ-HT-6d-B |  | 10,428,347 | 7,854,255 |
| H-6-C | LYQ-HT-6d-C |  | 11,176,986 | 8,403,410 |
| H-8-A | LYQ-HT-8d-A | heat treatment, 8 d | 13,788,114 | 10,372,169 |
| H-8-B | LYQ-HT-8d-B |  | 12,761,274 | 9,610,464 |
| H-8-C | LYQ-HT-8d-C |  | 10,633,762 | 8,081,794 |
| 0-0-A | LYQ-0d-A | 0d, is the harvest day | 10,660,022 | 8,091,921 |
| 0-0-B | LYQ-0d-B |  | 14,489,686 | 11,199,930 |
| 0-1-C | LYQ-0d-C |  | 15,661,005 | 12,084,062 |
| 0-1-A | LYQ-0OC-1d-A | 0 C 1d | 11,810,757 | 9,045,427 |
| 0-1-B | LYQ-0OC-1d-B |  | 15,804,608 | 11,969,502 |
| 0-1-C | LYQ-0OC-1d-C |  | 18,279,616 | 13,758,724 |
| 0-2-A | LYQ-0OC-2d-A | 0 C 2d | 14,830,488 | 11,283,188 |
| 0-2-B | LYQ-0OC-2d-B |  | 13,762,408 | 10,468,349 |
| 0-2-C | LYQ-0OC-2d-C |  | 7,544,147 | 5,678,467 |
| 0-4-A | LYQ-0OC-4d-A | 0 C 4d | 8,942,747 | 6,803,479 |
| 0-4-B | LYQ-0OC-4d-B |  | 12,551,575 | 9,604,055 |
| 0-4-C | LYQ-0OC-4d-C |  | 11,978,755 | 9,162,229 |
| 0-6-A | LYQ-0OC-6d-A | 0 C 6d | 13,271,117 | 10,240,305 |
| 0-6-B | LYQ-0OC-6d-B |  | 7,352,430 | 5,620,010 |
| 0-6-C | LYQ-0OC-6d-C |  | 8,464,027 | 6,473,579 |
| 0-8-A | LYQ-0OC-8d-A | 0 C 8d | 11,550,799 | 8,630,950 |
| 0-8-B | LYQ-0OC-8d-B |  | 6,606,212 | 5,116,111 |
| 0-8-C | LYQ-0OC-8d-C |  | 11,605,209 | 8,870,204 |

**Supplemental Table 2:** Genes differentially expressed in at least four time points in LTC or HT. There are 101 up-regulated and 79 down-regulated genes in LTC, and 50 and 13 in HT.

| Category | Gene-ID | Annotation |
| --- | --- | --- |
| LTC-up | UN05878 | 1-aminocyclopropane-1-carboxylate oxidase |
|  | UN42192 | Alpha-1,4-glucan-protein synthase [UDP-forming], putative |
|  | UN15616 | Amino acid permease |
|  | UN48825 | Amino acid permease |
|  | UN17693 | AP2-like ethylene-responsive transcription factor |
|  | UN32024 | Arginine and glutamate-rich protein 1-A |
|  | UN42765 | Auxin efflux carrier family protein |
|  | UN02018 | Auxin induced-like protein |
|  | UN34797 | auxin-regulated gene involved in organ size |
|  | UN29698 | B-cell receptor-associated 31-like |
|  | UN50380 | BHelix-loop-helix transcription factor |
|  | UN67992 | Bifunctional inhibitor/lipid-transfer protein/seed storage 2S albumin superfamily protein |
|  | UN49175 | Carbohydrate-binding X8 domain superfamily protein |
|  | UN67867 | Carbonyl reductase, putative |
|  | UN14684 | Cationic amino acid transporter, putative |
|  | UN00386 | CBL-interacting protein kinase 08 |
|  | UN26966 | CBL-interacting protein kinase 18 |
|  | UN42777 | Cc-nbs-lrr resistance protein |
|  | UN36097 | Cellulose synthase-like protein |
|  | UN19705 | Cytochrome P450, putative |
|  | UN68373 | Cytochrome P450, putative |
|  | UN48787 | Dehydrin 13 |
|  | UN68062 | Dihydrolipoamide S-acetyltransferase family protein |
|  | UN31458 | Double Clp-N motif-containing P-loop nucleoside triphosphate hydrolases superfamily protein |
|  | UN41845 | Enoyl-CoA hydratase/isomerase family protein |
|  | UN67905 | Enzyme of the cupin family protein |
|  | UN49989 | Eukaryotic aspartyl protease family protein |
|  | UN39722 | F-box domain containing protein |
|  | UN07984 | F-box protein PP2-B3 |
|  | UN22684 | formin homology 1 |
|  | UN15774 | Formin Homology 14 |
|  | UN15836 | Formin Homology 14 |
|  | UN51568 | Formin Homology 14 |
|  | UN49695 | Fructokinase-like protein 1 |
|  | UN37187 | GAI protein |
|  | UN33768 | Gibberellin 2-oxidase |
|  | UN68191 | Gibberellin 3-beta hydroxylase |
|  | UN31119 | Glucan endo-1,3-beta-glucosidase, putative |
|  | UN07811 | Glutaredoxin family protein |
|  | UN09461 | Golden 2-like 1 transcription factor |
|  | UN32936 | GTP-binding protein |
|  | UN06587 | high affinity nitrate transporter 2.7 |
|  | UN00628 | Histidine-rich glycoprotein |
|  | UN33380 | HXXXD-type acyl-transferase family protein |
|  | UN04646 | Inositol 1,3,4-trisphosphate 5/6-kinase family protein |
|  | UN40379 | Kinase, putative |
|  | UN40381 | Kinase, putative |
|  | UN23775 | Leucine rich repeat receptor kinase, putative |
|  | UN41939 | Leucine-rich repeat (LRR) family protein |
|  | UN49658 | Leucine-rich repeat (LRR) family protein |
|  | UN08943 | Leucine-rich repeat receptor kinase-like protein |
|  | UN23095 | Leucine-rich repeat receptor-like protein kinase |
|  | UN48841 | LOB domain protein |
|  | UN30109 | Major facilitator superfamily protein |
|  | UN31111 | MATE efflux family protein |
|  | UN29483 | Mitochondrial carrier protein, putative |
|  | UN32578 | Mitotic checkpoint protein bub3, putative |
|  | UN49625 | Monoglyceride lipase |
|  | UN22343 | Mucin-2 |
|  | UN21926 | NAC domain-containing protein |
|  | UN49062 | Outer mitochondrial membrane protein porin 1 |
|  | UN47664 | Oxysterol-binding protein, putative |
|  | UN12673 | Pectate lyase-like protein 1 |
|  | UN35268 | Pectinacetylesterase family protein |
|  | UN50203 | Pentatricopeptide repeat-containing family protein |
|  | UN16523 | Pentatricopeptide repeat-containing protein |
|  | UN48814 | Pentatricopeptide repeat-containing protein, putative |
|  | UN36959 | Pheromone-processing carboxypeptidase KEX1 |
|  | UN49302 | proline extensin-like receptor kinase 1 |
|  | UN03887 | Prolyl endopeptidase |
|  | UN45161 | Protein FAM116A |
|  | UN15382 | Protein kinase |
|  | UN67938 | Protein LHCP TRANSLOCATION DEFECT |
|  | UN31466 | Protein of unknown function, DUF584 |
|  | UN37213 | Protein phosphatase 2c, putative |
|  | UN09417 | Ribulose bisphosphate carboxylase/oxygenase activase family protein |
|  | UN29190 | Ring finger protein, putative |
|  | UN30096 | RING/U-box superfamily protein |
|  | UN68158 | RING/U-box superfamily protein |
|  | UN30230 | RNA binding protein, putative |
|  | UN68030 | RNA-binding family protein |
|  | UN11470 | Scarecrow transcription factor family protein |
|  | UN40211 | Serine/threonine protein phosphatase 2A activator |
|  | UN11170 | serine-rich protein-related |
|  | UN48979 | Serine-threonine protein kinase, plant-type, putative |
|  | UN22344 | Sex-determining region Y protein |
|  | UN20597 | SKI family transcriptional corepressor 2 |
|  | UN51081 | TCP family transcription factor family protein |
|  | UN40207 | Transducin/WD40 repeat-like superfamily protein |
|  | UN17927 | Transposon Ty1-H Gag-Pol polyprotein |
|  | UN06276 | tRNA dimethylallyltransferase |
|  | UN46565 | Ubiquitin-conjugating enzyme h, putative |
|  | UN49285 | U-box domain-containing family protein |
|  | UN35156 | UDP-glucuronate decarboxylase protein 1 |
|  | UN52569 | UDP-glycosyltransferase 1 |
|  | UN16620 | UDP-sugar transporter, putative |
|  | UN16775 | Vegetative cell wall protein gp1 |
|  | UN36836 | WRKY DNA-binding protein 57 |
|  | UN10623 | Zinc finger family protein |
|  | UN49058 | Zinc finger homeobox protein 4 |
|  | UN07783 | Zinc transporter 4 |
| LTC-down | UN49134 | 1-aminocyclopropane-1-carboxylate oxidase, putative |
|  | UN33644 | Acyl-CoA thioesterase, putative |
|  | UN35881 | Acyl-protein thioesterase, putative |
|  | UN34908 | Adenine nucleotide alpha hydrolases-like protein |
|  | UN38262 | Alcohol dehydrogenase |
|  | UN68301 | Alcohol dehydrogenase, putative |
|  | UN03052 | Alpha 1,3 fucosyltransferase |
|  | UN40915 | Ankyrin repeat family protein |
|  | UN33535 | AP2-like ethylene-responsive transcription factor |
|  | UN68212 | arabinogalactan protein 1 |
|  | UN29442 | Calcium-dependent lipid-binding (CaLB domain) family protein |
|  | UN07225 | Calcium-transporting ATPase 8 family protein |
|  | UN15368 | Calmodulin-like family protein |
|  | UN48658 | Cation-transporting atpase plant, putative |
|  | UN08030 | Chaperone protein dnaJ |
|  | UN52911 | Chaperone protein dnaJ |
|  | UN07944 | Chorismate mutase 01 |
|  | UN09922 | Constitutive expressor of pathogenesis related genes 5 copy 1 |
|  | UN14789 | cytochrome BC1 synthesis |
|  | UN22184 | DNA-directed RNA polymerase II subunit rpb1 |
|  | UN40308 | Dof zinc finger protein |
|  | UN14964 | Double Clp-N motif-containing P-loop nucleoside triphosphate hydrolases superfamily protein |
|  | UN17068 | Early nodulin, putative |
|  | UN67975 | Endosomal targeting BRO1-like domain-containing protein |
|  | UN27169 | Extensin, proline-rich protein, putative |
|  | UN49264 | Galactinol synthase |
|  | UN21207 | Germin-like protein 6 |
|  | UN33287 | Glutamate decarboxylase, putative |
|  | UN33349 | glutamine dumper 1 |
|  | UN15780 | Glutathione S-transferase-like protein |
|  | UN53849 | Glycerol-3-phosphate acyltransferase |
|  | UN68513 | Glycine-rich protein |
|  | UN23387 | Heat stress transcription factor C-2b |
|  | UN12676 | Histone H2B |
|  | UN12680 | Histone H2B |
|  | UN45909 | Importin subunit alpha-8 |
|  | UN31480 | Kinase family protein |
|  | UN54503 | Kinase family protein |
|  | UN32514 | Late embryogenesis abundant (LEA) hydroxyproline-rich glycoprotein family |
|  | UN22561 | Leucine-rich repeat (LRR) family protein |
|  | UN16797 | Leucine-rich repeat (LRR) family protein |
|  | UN22352 | Lipid transfer protein |
|  | UN42267 | Lipoxygenase |
|  | UN48968 | Major facilitator superfamily protein |
|  | UN41965 | Mediator of RNA polymerase II transcription subunit 12 |
|  | UN19253 | Myb domain protein 97 |
|  | UN26543 | NAD(P)H dehydrogenase (quinone) |
|  | UN31909 | Nbs-lrr resistance protein |
|  | UN34681 | Nbs-lrr resistance protein |
|  | UN23269 | Phosphate transporter PHO1-3 |
|  | UN13139 | Phosphatidylinositol N-acetyglucosaminlytransferase subunit P-related |
|  | UN25602 | Phospholipase D |
|  | UN16769 | Plant calmodulin-binding protein-related |
|  | UN44325 | Plant protein of unknown function (DUF946) |
|  | UN10545 | Pleiotropic drug resistance, ABC transporter family protein |
|  | UN09031 | Potassium transporter 5 family protein |
|  | UN32909 | Prephenate dehydratase family protein |
|  | UN48914 | Proline-rich protein |
|  | UN22063 | Protein of unknown function, DUF538 |
|  | UN35443 | Protein phosphatase 2c, putative |
|  | UN50077 | Pyruvate decarboxylase family protein |
|  | UN37722 | Radical-induced cell death 1 |
|  | UN39827 | RAN BINDING protein 1 |
|  | UN08143 | Reverse transcriptase |
|  | UN35182 | RING finger family protein |
|  | UN32872 | Ring finger protein, putative |
|  | UN10556 | RING/FYVE/PHD zinc finger superfamily protein |
|  | UN12094 | Rubber elongation factor protein (REF) |
|  | UN27314 | smr (Small MutS Related) domain-containing protein |
|  | UN49801 | Sorbitol dehydrogenase |
|  | UN44508 | Transcription elongation factor A protein |
|  | UN35957 | Transcription initiation factor IIB |
|  | UN22185 | Translation initiation factor IF-2 |
|  | UN35676 | Ubiquitin carboxyl-terminal hydrolase |
|  | UN26748 | UDP-Glycosyltransferase superfamily protein |
|  | UN49923 | Yellow stripe-like transporter 11 |
|  | UN37002 | Zinc finger A20 and AN1 domain-containing stress-associated protein |
|  | UN11753 | Zinc finger protein CONSTANS-like protein |
|  | UN12188 | Zinc-binding dehydrogenase family protein |
| HT-up | UN49691 | Adenine nucleotide alpha hydrolases-like protein |
|  | UN50398 | Adhesive/proline-rich protein |
|  | UN06411 | Alcohol dehydrogenase |
|  | UN49147 | Aquaporin |
|  | UN33371 | Ascorbate peroxidase |
|  | UN48884 | BAG family molecular chaperone regulator 6 |
|  | UN28263 | Blue copper protein, putative |
|  | UN50743 | Blue copper-like protein |
|  | UN48596 | BZIP transcription factor |
|  | UN31898 | Calcyclin-binding protein, putative |
|  | UN00386 | CBL-interacting protein kinase 08 |
|  | UN05134 | Chaperone clpb, putative |
|  | UN49100 | Circumsporozoite protein |
|  | UN32993 | Ethylene-dependent gravitropism-deficient and yellow-green-like 3 protein |
|  | UN51053 | extensin proline-rich 1 |
|  | UN11136 | Fasciclin-like arabinogalactan protein 1-like protein |
|  | UN48831 | Galactinol synthase 6 |
|  | UN68191 | Gibberellin 3-beta hydroxylase |
|  | UN49418 | hydroxyproline-rich glycoprotein family protein |
|  | UN29745 | Lactoylglutathione lyase, putative |
|  | UN30754 | leucine-rich repeat/extensin 2 |
|  | UN49342 | Nuclear transcription factor Y subunit gamma |
|  | UN38113 | Peptidyl-prolyl cis-trans isomerase |
|  | UN42088 | Peptidyl-prolyl cis-trans isomerase |
|  | UN68005 | Phosphosulfolactate synthase-related protein |
|  | UN14354 | proline-rich extensin-like receptor kinase 10 |
|  | UN30230 | RNA binding protein, putative |
|  | UN40514 | Serine protease |
|  | UN20633 | Short-chain alcohol dehydrogenase |
|  | UN23100 | Thiamine thiazole synthase, chloroplastic |
|  | UN13450 | Transposon Ty1-PL Gag-Pol polyprotein |
|  | UN35156 | UDP-glucuronate decarboxylase protein 1 |
|  | UN49973 | UNUSUAL FLORAL ORGANS family protein |
|  | UN48950 | 17.4 kDa class I heat shock family protein |
|  | UN48983 | 17.5 kDa class I heat shock protein |
|  | UN37354 | 17.5 kDa class II heat shock protein |
|  | UN26798 | 18.2 kDa class I heat shock family protein |
|  | UN48894 | 18.2 kDa class I heat shock family protein |
|  | UN29318 | 22.0 kDa class IV heat shock protein |
|  | UN23641 | 22.7 kDa class IV heat shock protein |
|  | UN23051 | 70 kDa heat shock protein |
|  | UN31228 | 70 kDa heat shock protein |
|  | UN35084 | 70 kDa heat shock protein |
|  | UN13193 | 70-kDa heat shock protein |
|  | UN29376 | Heat shock 70 kDa protein 1 |
|  | UN05128 | Heat shock 70 kDa protein C |
|  | UN68121 | Heat shock 70 kDa protein, putative |
|  | UN31360 | Heat shock protein, putative |
|  | UN33977 | Heat shock protein, putative |
|  | UN48868 | Heat-shock protein, putative |
| HT-down | UN08876 | 5'-methylthioadenosine/S-adenosylhomocysteine nucleosidase |
|  | UN14792 | O-methyltransferase-like protein |
|  | UN20167 | Triose phosphate/phosphate translocator |
|  | UN21207 | Germin-like protein 6 |
|  | UN28125 | Myosin-10 |
|  | UN34934 | TRICHOME BIREFRINGENCE-LIKE 39 |
|  | UN34989 | Pirin-like protein |
|  | UN43022 | Serine carboxypeptidase, putative |
|  | UN44122 | Alpha-glucosidase, putative |
|  | UN48804 | Gibberellin-regulated protein 5 |
|  | UN53812 | Xyloglucan endotransglucosylase/hydrolase 1 |
|  | UN68166 | Laccase, putative |
|  | UN68301 | Alcohol dehydrogenase, putative |

**Supplemental Table 3:** Expression (RPKM) of genes differentially expressed in at least four time points in both LTC and HT.

|  | Gene-ID | annotation | treatment | Day-1 | Day-2 | Day-4 | Day-6 | Day-8 |
| --- | --- | --- | --- | --- | --- | --- | --- | --- |
| up | UN30230 | RNA binding protein, putative | 0^o^C | 15.12 | 12.35 | 15.10 | 14.52 | 12.64 |
|  |  |  | LTC | 33.99 | 31.82 | 33.45 | 34.90 | 44.39 |
|  |  |  | HT | 28.04 | 28.18 | 53.27 | 34.59 | 42.79 |
|  | UN00386 | CBL-interacting protein kinase 08 | 0^o^C | 3.55 | 7.72 | 4.20 | 0.001 | 2.84 |
|  |  |  | LTC | 51.19 | 51.24 | 54.18 | 42.05 | 35.42 |
|  |  |  | HT | 14.28 | 30.30 | 44.52 | 26.68 | 42.14 |
|  | UN68191 | Gibberellin 3-beta hydroxylase | 0^o^C | 100.3 | 70.42 | 68.07 | 60.71 | 84.68 |
|  |  |  | LTC | 110.2 | 171.0 | 173.5 | 204.0 | 239.7 |
|  |  |  | HT | 92.26 | 214.7 | 257.8 | 192.2 | 175.7 |
|  | UN35156 | UDP-glucuronate decarboxylase protein 1 | 0^o^C | 15.85 | 19.16 | 33.75 | 37.78 | 38.51 |
|  |  |  | LTC | 63.24 | 55.21 | 74.08 | 75.50 | 58.75 |
|  |  |  | HT | 31.46 | 42.74 | 81.55 | 72.23 | 78.48 |
| down | UN68301 | Alcohol dehydrogenase, putative | 0^o^C | 38.91 | 41.41 | 25.84 | 29.52 | 21.20 |
|  |  |  | LTC | 16.99 | 14.98 | 10.02 | 8.36 | 9.71 |
|  |  |  | HT | 14.70 | 11.42 | 9.58 | 7.51 | 7.69 |
|  | UN21207 | Germin-like protein 6 | 0^o^C | 12.55 | 20.80 | 17.63 | 12.05 | 6.39 |
|  |  |  | LTC | 13.27 | 6.25 | 3.62 | 0.96 | 0.29 |
|  |  |  | HT | 9.91 | 8.20 | 3.64 | 1.36 | 1.21 |

**Supplemental Table 4**: Top 100 genes selected using the entropy weight method.

| index | ID (LTC) | annotation | ID (HT) | annotation |
| --- | --- | --- | --- | --- |
| 1 | UN00386 | CBL-interacting protein kinase 08 | UN29318 | 22.0 kDa class IV heat shock protein |
| 2 | UN49801 | Sorbitol dehydrogenase | UN48950 | 17.4 kDa class I heat shock family protein |
| 3 | UN29442 | Calcium-dependent lipid-binding (CaLB domain) family protein | UN48983 | 17.5 kDa class I heat shock protein |
| 4 | UN41845 | Enoyl-CoA hydratase/isomerase family protein | UN37354 | 17.5 kDa class II heat shock protein |
| 5 | UN26748 | UDP-Glycosyltransferase superfamily protein | UN14354 | proline-rich extensin-like receptor kinase 10 |
| 6 | UN12094 | Rubber elongation factor protein (REF) | UN48868 | Heat-shock protein, putative |
| 7 | UN22184 | DNA-directed RNA polymerase II subunit rpb1 | UN48894 | 18.2 kDa class I heat shock family protein |
| 8 | UN12676 | Histone H2B | UN38113 | Peptidyl-prolyl cis-trans isomerase |
| 9 | UN08030 | Chaperone protein dnaJ | UN23641 | 22.7 kDa class IV heat shock protein |
| 10 | UN48914 | Proline-rich protein | UN35084 | 70 kDa heat shock protein |
| 11 | UN22136 | Polyubiquitin 10 protein | UN26798 | 18.2 kDa class I heat shock family protein |
| 12 | UN09487 | Haloacid dehalogenase-like hydrolase (HAD) superfamily protein | UN48884 | BAG family molecular chaperone regulator 6 |
| 13 | UN22185 | Translation initiation factor IF-2 | UN48804 | Gibberellin-regulated protein 5 |
| 14 | UN48841 | LOB domain protein | UN68166 | Laccase, putative |
| 15 | UN33768 | Gibberellin 2-oxidase | UN31360 | Heat shock protein, putative |
| 16 | UN48814 | Pentatricopeptide repeat-containing protein, putative | UN13193 | 70-kDa heat shock protein |
| 17 | UN29487 | Polyubiquitin | UN09487 | Haloacid dehalogenase-like hydrolase (HAD) superfamily protein |
| 18 | UN03887 | Prolyl endopeptidase | UN22256 | Haloacid dehalogenase-like hydrolase family protein, putative, expressed |
| 19 | UN32909 | Prephenate dehydratase family protein | UN08030 | Chaperone protein dnaJ |
| 20 | UN40381 | Kinase, putative | UN68223 | Kunitz trypsin inhibitor |
| 21 | UN31119 | Glucan endo-1,3-beta-glucosidase, putative | UN68222 | Beta-D-xylosidase |
| 22 | UN34931 | Ribose 5-phosphate isomerase family protein | UN26747 | Leucine-rich repeat (LRR) family protein |
| 23 | UN09479 | Ubiquitin | UN00628 | Histidine-rich glycoprotein |
| 24 | UN22344 | Sex-determining region Y protein | UN32515 | Late embryogenesis abundant (LEA) hydroxyproline-rich glycoprotein family |
| 25 | UN33495 | Basic-leucine zipper (bZIP) transcription factor family protein | UN48831 | Galactinol synthase 6 |
| 26 | UN49085 | Histidine decarboxylase | UN68191 | Gibberellin 3-beta hydroxylase |
| 27 | UN48979 | Serine-threonine protein kinase, plant-type, putative | UN48850 | Late embryogenesis abundant (LEA) hydroxyproline-rich glycoprotein family |
| 28 | UN15370 | Calcium-binding protein 5 | UN49801 | Sorbitol dehydrogenase |
| 29 | UN15368 | Calmodulin-like family protein | UN48799 | leucine-rich repeat/extensin 2 |
| 30 | UN22256 | Haloacid dehalogenase-like hydrolase family protein, putative, expressed | UN31772 | Metallothionein-like protein |
| 31 | UN68314 | Homeobox-leucine zipper protein HAT14, putative | UN49342 | Nuclear transcription factor Y subunit gamma |
| 32 | UN40207 | Transducin/WD40 repeat-like superfamily protein | UN03042 | Actin |
| 33 | UN68166 | Laccase, putative | UN68159 | Late embryogenesis abundant (LEA) hydroxyproline-rich glycoprotein |
| 34 | UN33497 | Basic-leucine zipper (bZIP) transcription factor family protein | UN26746 | Leucine-rich repeat (LRR) family protein |
| 35 | UN22343 | Mucin-2 | UN09439 | leucine-rich repeat/extensin 1 |
| 36 | UN36959 | Pheromone-processing carboxypeptidase KEX1 | UN02886 | RNA-binding protein |
| 37 | UN49058 | Zinc finger homeobox protein 4 | UN68158 | RING/U-box superfamily protein |
| 38 | UN31458 | Double Clp-N motif-containing P-loop nucleoside triphosphate hydrolases superfamily protein | UN12246 | 70-kDa heat shock protein |
| 39 | UN33380 | HXXXD-type acyl-transferase family protein | UN48834 | Bidirectional sugar transporter N3 |
| 40 | UN22245 | hydroxyproline-rich glycoprotein family protein | UN50135 | NADH-ubiquinone oxidoreductase 20 kDa subunit, mitochondrial |
| 41 | UN38262 | Alcohol dehydrogenase | UN68192 | Gibberellin 3-beta hydroxylase |
| 42 | UN07811 | Glutaredoxin family protein | UN33633 | 4-hydroxyphenylpyruvate dioxygenase |
| 43 | UN48837 | BZIP transcription factor family protein | UN48824 | lectin protein kinase family protein |
| 44 | UN49175 | Carbohydrate-binding X8 domain superfamily protein | UN22125 | C2 domain-containing protein |
| 45 | UN51203 | Leucine-rich repeat extensin-like protein 3 | UN48794 | Pectinesterase |
| 46 | UN12188 | Zinc-binding dehydrogenase family protein | UN47237 | 1-aminocyclopropane-1-carboxylate oxidase |
| 47 | UN68223 | Kunitz trypsin inhibitor | UN35486 | Protein translation factor SUI1 |
| 48 | UN68158 | RING/U-box superfamily protein | UN20814 | Sulfate transporter |
| 49 | UN06276 | tRNA dimethylallyltransferase | UN68164 | Phytosulfokines 6 family protein |
| 50 | UN15836 | Formin Homology 14 | UN48957 | CG32676 |
| 51 | UN37004 | A20/AN1-like zinc finger family protein | UN16416 | Laccase family protein |
| 52 | UN50026 | Senescence-inducible chloroplast stay-green protein | UN12676 | Histone H2B |
| 53 | UN26912 | Universal stress protein A-like protein | UN41845 | Enoyl-CoA hydratase/isomerase family protein |
| 54 | UN35268 | Pectinacetylesterase family protein | UN26714 | Lipoxygenase |
| 55 | UN36944 | CBL-interacting protein kinase 08 | UN26980 | Ubiquitin conjugating enzyme 9 |
| 56 | UN48787 | Dehydrin 13 | UN48979 | Serine-threonine protein kinase, plant-type, putative |
| 57 | UN42192 | Alpha-1,4-glucan-protein synthase [UDP-forming], putative | UN31229 | Late embryogenesis abundant protein (LEA) family protein |
| 58 | UN48833 | Beta-carotene hydroxylase | UN15774 | Formin Homology 14 |
| 59 | UN48803 | Polyphenol oxidase | UN33495 | Basic-leucine zipper (bZIP) transcription factor family protein |
| 60 | UN07783 | Zinc transporter 4 | UN26753 | Proline rich protein |
| 61 | UN43474 | Alkaline alpha galactosidase I family protein | UN33354 | Calmodulin-binding transcription activator 2 |
| 62 | UN06587 | high affinity nitrate transporter 2.7 | UN49100 | Circumsporozoite protein |
| 63 | UN48804 | Gibberellin-regulated protein 5 | UN31026 | RING finger protein |
| 64 | UN48825 | Amino acid permease | UN68314 | Homeobox-leucine zipper protein HAT14, putative |
| 65 | UN49625 | Monoglyceride lipase | UN49058 | Zinc finger homeobox protein 4 |
| 66 | UN68071 | Mitogen-activated protein kinase kinase 1 | UN40514 | Serine protease |
| 67 | UN34479 | Senescence-associated protein DIN1, putative | UN35514 | Alanine aminotransferase |
| 68 | UN32514 | Late embryogenesis abundant (LEA) hydroxyproline-rich glycoprotein family | UN09461 | Golden 2-like 1 transcription factor |
| 69 | UN01157 | Uncharacterised conserved protein (UCP030210) | UN45161 | Protein FAM116A |
| 70 | UN19705 | Cytochrome P450, putative | UN10032 | Xyloglucan endotransglucosylase/hydrolase 7 |
| 71 | UN26814 | Polyubiquitin | UN42192 | Alpha-1,4-glucan-protein synthase [UDP-forming], putative |
| 72 | UN49140 | B-box zinc finger family protein | UN40072 | Sigma factor sigb regulation protein rsbq, putative |
| 73 | UN17068 | Early nodulin, putative | UN52077 | Protein ea47 |
| 74 | UN34908 | Adenine nucleotide alpha hydrolases-like protein | UN68152 | Leucine-rich repeat (LRR) family protein |
| 75 | UN33119 | Senescence-inducible chloroplast stay-green protein 2 | UN49166 | Succinyl-CoA ligase [GDP-forming] subunit alpha-2, mitochondrial, putative |
| 76 | UN50267 | nucleolin like 1 | UN00648 | Malic enzyme |
| 77 | UN10032 | Xyloglucan endotransglucosylase/ hydrolase 7 | UN48857 | leucine-rich repeat/extensin 2 |
| 78 | UN48858 | Polyphenol oxidase | UN50204 | HD domain class transcription factor |
| 79 | UN16416 | Laccase family protein | UN36548 | Expansin 2 |
| 80 | UN49264 | Galactinol synthase | UN35156 | UDP-glucuronate decarboxylase protein 1 |
| 81 | UN41939 | Leucine-rich repeat (LRR) family protein | UN39638 | DAG protein |
| 82 | UN48823 | Ethylene responsive transcription factor 2a | UN48861 | Basic-leucine zipper (bZIP) transcription factor family protein |
| 83 | UN29190 | Ring finger protein, putative | UN32221 | Expansin-like protein |
| 84 | UN00931 | Beta-amylase | UN22344 | Sex-determining region Y protein |
| 85 | UN09997 | Polyubiquitin 10 protein | UN43915 | 60 kDa chaperonin 1 |
| 86 | UN49013 | Galactinol synthase | UN22245 | hydroxyproline-rich glycoprotein family protein |
| 87 | UN16720 | Late embryogenesis abundant (LEA) hydroxyproline-rich glycoprotein family | UN41276 | Apple fruit acidity-related protein |
| 88 | UN49062 | Outer mitochondrial membrane protein porin 1 | UN68063 | Adhesive/proline-rich protein |
| 89 | UN68191 | Gibberellin 3-beta hydroxylase | UN49093 | Leucine-rich repeat (LRR) family protein |
| 90 | UN49134 | 1-aminocyclopropane-1-carboxylate oxidase, putative | UN41548 | Formin Homology 14 |
| 91 | UN48830 | Glucan endo-1,3-beta-glucosidase 4 | UN15104 | Purine permease-related family protein |
| 92 | UN36017 | ABC transporter | UN32271 | Prefoldin subunit |
| 93 | UN39167 | Ribosomal protein L34e superfamily protein | UN37031 | Early nodulin 16, putative |
| 94 | UN12169 | Histone H2B | UN49147 | Aquaporin |
| 95 | UN39955 | Apoplastic invertase | UN46899 | Transcription factor SPT20 homolog |
| 96 | UN49421 | V-type proton ATPase subunit G | UN00386 | CBL-interacting protein kinase 08 |
| 97 | UN34543 | Phi-1-like phosphate-induced protein | UN49468 | ATP sulfurylase |
| 98 | UN45161 | Protein FAM116A | UN34095 | Aquaporin |
| 99 | UN31155 | Pollen Ole e 1 allergen and extensin family protein | UN68373 | Cytochrome P450, putative |
| 100 | UN35962 | F-box family protein | UN46901 | Transcription factor SPT20 homolog |

**Supplemental Table 5:** Expression (RPKM) of the top 10 genes selected using the entropy weight method (heat shock proteins were excluded in HT).

|  | Gene ID | annotation | Treatment | Day-1 | Day-2 | Day-4 | Day-6 | Day-8 |
| --- | --- | --- | --- | --- | --- | --- | --- | --- |
| LTC | UN00386 | CBL-interacting protein kinase 08 | 0^o^C | 3.55 | 7.72 | 4.20 | 0.001 | 2.84 |
|  |  |  | LTC | 51.19 | 51.24 | 54.18 | 42.05 | 35.42 |
|  |  |  | HT | 14.28 | 30.30 | 44.52 | 26.68 | 42.14 |
|  | UN49801 | Sorbitol dehydrogenase | 0^o^C | 198.2 | 427.5 | 112.8 | 94.51 | 50.78 |
|  |  |  | LTC | 43.96 | 16.89 | 11.11 | 10.90 | 100.4 |
|  |  |  | HT | 74.30 | 230.4 | 25.77 | 25.54 | 22.76 |
|  | UN29442 | Calcium-dependent lipid-binding (CaLB domain) family protein | 0^o^C | 104.3 | 119.0 | 254.2 | 434.6 | 411.6 |
|  |  |  | LTC | 50.08 | 47.29 | 51.01 | 67.96 | 108.0 |
|  |  |  | HT | 157.6 | 151.7 | 414.0 | 481.4 | 500.5 |
|  | UN41845 | Enoyl-CoA hydratase/isomerase family protein | 0^o^C | 70.15 | 29.62 | 39.12 | 35.59 | 23.86 |
|  |  |  | LTC | 151.1 | 131.9 | 178.8 | 140.0 | 88.47 |
|  |  |  | HT | 77.84 | 58.47 | 96.32 | 112.2 | 123.9 |
|  | UN26748 | UDP-Glycosyltransferase superfamily protein | 0^o^C | 177.4 | 377.4 | 878.5 | 1227 | 1127 |
|  |  |  | LTC | 98.81 | 133.9 | 230.6 | 203.1 | 353.3 |
|  |  |  | HT | 201.3 | 283.5 | 1050 | 1219 | 1129 |
|  | UN12094 | Rubber elongation factor protein (REF) | 0^o^C | 64.70 | 79.59 | 191.1 | 233.4 | 259.5 |
|  |  |  | LTC | 27.48 | 32.31 | 45.46 | 44.74 | 59.89 |
|  |  |  | HT | 58.90 | 57.77 | 152.2 | 157.4 | 163.3 |
|  | UN22184 | DNA-directed RNA polymerase II subunit rpb1 | 0^o^C | 508.1 | 935.4 | 1510 | 2608 | 2565 |
|  |  |  | LTC | 453.3 | 318.7 | 350.6 | 305.4 | 807.5 |
|  |  |  | HT | 838.5 | 918.7 | 1853 | 1413 | 1822 |
|  | UN12676 | Histone H2B | 0^o^C | 81.76 | 111.1 | 116.4 | 105.9 | 92.62 |
|  |  |  | LTC | 41.00 | 28.55 | 29.58 | 23.63 | 26.32 |
|  |  |  | HT | 125.3 | 87.91 | 32.94 | 34.96 | 26.79 |
|  | UN08030 | Chaperone protein dnaJ | 0^o^C | 63.88 | 77.52 | 154.9 | 249.9 | 207.8 |
|  |  |  | LTC | 25.12 | 33.74 | 24.29 | 51.29 | 81.75 |
|  |  |  | HT | 19.28 | 23.75 | 48.24 | 174.0 | 109.9 |
|  | UN48914 | Proline-rich protein | 0^o^C | 257.8 | 301.4 | 318.9 | 268.5 | 156.9 |
|  |  |  | LTC | 217.3 | 135.8 | 60.92 | 26.81 | 50.77 |
|  |  |  | HT | 482.3 | 240.2 | 179.4 | 68.07 | 373.3 |
| HT | UN14354 | proline-rich extensin-like receptor kinase 10 | 0^o^C | 21.77 | 12.52 | 14.24 | 12.91 | 19.86 |
|  |  |  | LTC | 8.99 | 7.39 | 7.84 | 6.51 | 11.70 |
|  |  |  | HT | 235.7 | 242.1 | 268.4 | 228.0 | 271.7 |
|  | UN38113 | Peptidyl-prolyl cis-trans isomerase | 0^o^C | 38.38 | 39.76 | 35.66 | 33.70 | 44.58 |
|  |  |  | LTC | 29.46 | 28.14 | 30.22 | 32.15 | 33.63 |
|  |  |  | HT | 655.8 | 518.9 | 246.4 | 142.5 | 142.5 |
|  | UN48884 | BAG family molecular chaperone regulator 6 | 0^o^C | 119.6 | 55.56 | 113.4 | 88.67 | 65.72 |
|  |  |  | LTC | 71.17 | 72.94 | 37.84 | 69.49 | 69.03 |
|  |  |  | HT | 286.7 | 266.5 | 275.9 | 303.7 | 284.6 |
|  | UN48804 | Gibberellin-regulated protein 5 | 0^o^C | 2077 | 1753 | 1594 | 1153 | 615.2 |
|  |  |  | LTC | 1531 | 1113 | 455.2 | 234.2 | 345.8 |
|  |  |  | HT | 1027 | 1005 | 413.2 | 268.1 | 202.5 |
|  | UN68166 | Laccase, putative | 0^o^C | 1968 | 1687 | 955.6 | 408.7 | 289.2 |
|  |  |  | LTC | 2607 | 1628 | 233.1 | 60.75 | 40.81 |
|  |  |  | HT | 642.7 | 669.7 | 336.5 | 150.4 | 114.7 |
|  | UN09487 | Haloacid dehalogenase-like hydrolase (HAD) superfamily protein | 0^o^C | 939.1 | 944.0 | 644.5 | 488.5 | 343.8 |
|  |  |  | LTC | 829.9 | 581.5 | 167.1 | 74.03 | 49.79 |
|  |  |  | HT | 511.8 | 499.4 | 272.9 | 212.2 | 142.4 |
|  | UN22256 | Haloacid dehalogenase-like hydrolase family protein, putative, expressed | 0^o^C | 521.1 | 549.4 | 323.3 | 275.6 | 180.4 |
|  |  |  | LTC | 497.1 | 355.7 | 93.68 | 40.53 | 35.14 |
|  |  |  | HT | 297.8 | 219.4 | 149.5 | 127.52 | 69.30 |
|  | UN08030 | Chaperone protein dnaJ | 0^o^C | 63.88 | 77.52 | 154.9 | 249.9 | 207.8 |
|  |  |  | LTC | 25.12 | 33.74 | 24.29 | 51.29 | 81.75 |
|  |  |  | HT | 19.28 | 23.75 | 48.24 | 174.0 | 109.9 |
|  | UN68223 | Kunitz trypsin inhibitor | 0^o^C | 234.1 | 155.0 | 138.5 | 313.1 | 503.7 |
|  |  |  | LTC | 87.24 | 51.51 | 40.75 | 103.6 | 407.0 |
|  |  |  | HT | 41.55 | 29.52 | 154.9 | 493.7 | 275.0 |
|  | UN68222 | Beta-D-xylosidase | 0^o^C | 634.4 | 265.7 | 175.3 | 460.7 | 622.2 |
|  |  |  | LTC | 153.4 | 293.4 | 610.3 | 758.5 | 1393 |
|  |  |  | HT | 242.3 | 193.8 | 418.1 | 1437 | 500.6 |

**Supplemental Table 6:** Gene-specific oligonucleotide primers for real-time PCR analysis

| Gene | Forward primer (5’ to 3’) | Reverse primer (5’ to 3’) |
| --- | --- | --- |
| *EjCAD3* | CTCCCTAAAATGATATGAGGAATCG | CACTCCCATCTTAACAGCCAAAA |
| COMT (*UN14792*) | TTAAGCCGGAGACTGACCTATCT | GATTCCTGACCCAAAATTAGCGG |
| HCT (*UN28739*) | GATTCCTGACCCAAAATTAGCGG | ACCAGGATAAACTTCTCACGCAT |
